# Architectural Dynamics of CaMKII-Actin Networks

**DOI:** 10.1016/j.bpj.2018.11.006

**Published:** 2018-11-10

**Authors:** Shahid Khan, Kenneth H. Downing, Justin E. Molloy

**Affiliations:** 1 Molecular Biology Consortium, Lawrence Berkeley National Laboratory, Berkeley, California; 2 Molecular Biophysics and Integrated Bioimaging, Lawrence Berkeley National Laboratory, Berkeley, California; 3 The Francis Crick Institute, London, United Kingdom

## Abstract

Calcium-calmodulin-dependent kinase II (CaMKII) has an important role in dendritic spine remodeling upon synaptic stimulation. Using fluorescence video microscopy and image analysis, we investigated the architectural dynamics of rhodamine-phalloidin stabilized filamentous actin (F-actin) networks cross-linked by CaMKII. We used automated image analysis to identify F-actin bundles and crossover junctions and developed a dimensionless metric to characterize network architecture. Similar networks were formed by three different CaMKII species with a 10-fold length difference in the linker region between the kinase domain and holoenzyme hub, implying linker length is not a primary determinant of F-actin cross-linking. Electron micrographs showed that at physiological molar ratios, single CaMKII holoenzymes cross-linked multiple F-actin filaments at random, whereas at higher CaMKII/F-actin ratios, filaments bundled. Light microscopy established that the random network architecture resisted macromolecular crowding with polyethylene glycol and blocked ATP-powered compaction by myosin-II miniature filaments. Importantly, the networks disassembled after the addition of calcium-calmodulin and were then spaced within 3 min into compacted foci by myosin motors or more slowly (30 min) aggregated by crowding. Single-molecule total internal reflection fluorescence microscopy showed CaMKII dissociation from surface-immobilized globular actin exhibited a monoexponential dwell-time distribution, whereas CaMKII bound to F-actin networks had a long-lived fraction, trapped at crossover junctions. Release of CaMKII from F-actin, triggered by calcium-calmodulin, was too rapid to measure with flow-cell exchange (<20 s). The residual bound fraction was reduced substantially upon addition of an N-methyl-D-aspartate receptor peptide analog but not ATP. These results provide mechanistic insights to CaMKII-actin interactions at the collective network and single-molecule level. Our findings argue that CaMKII-actin networks in dendritic spines maintain spine size against physical stress. Upon synaptic stimulation, CaMKII is disengaged by calcium-calmodulin, triggering network disassembly, expansion, and subsequent compaction by myosin motors with kinetics compatible with the times recorded for the poststimulus changes in spine volume.

## Introduction

The calcium-calmodulin-dependent kinase II (CaMKII) is a multifunctional, dodecameric kinase assembly. It is ubiquitous in the animal kingdom and has key roles in learning and cardiovascular function. The role in learning has been studied in vertebrate rat models of memory (1, 2), and its effects on dendritic morphology and synaptic localization in the nematode *Caenorhabditis elegans* have been described (3). CaMKII is important both in long-term potentiation (LTP) and long-term depression, with the biochemical mechanisms best understood for LTP in hippocampal neurons (4). A direct structural role of CaMKII in dendritic spines has been proposed (5, 6, 7), consistent with its high (>0.1 mM) abundance (8) and actin-binding affinity as well as its kinase activity. The dendritic spine cytoskeleton is composed overwhelmingly of filamentous actin (F-actin). Actin binding by CaMKII and its abrogation by calcium-bound calmodulin (i.e., calcium-calmodulin) is central to spine remodeling during early LTP (9, 10). Here, we characterize the interactions of CaMKII in synthetic F-actin networks (i.e., CaMKII-actin networks) to better understand the structural role of CaMKII in maintenance and remodeling of the dendritic spine cytoskeleton.

**Figure 1:**
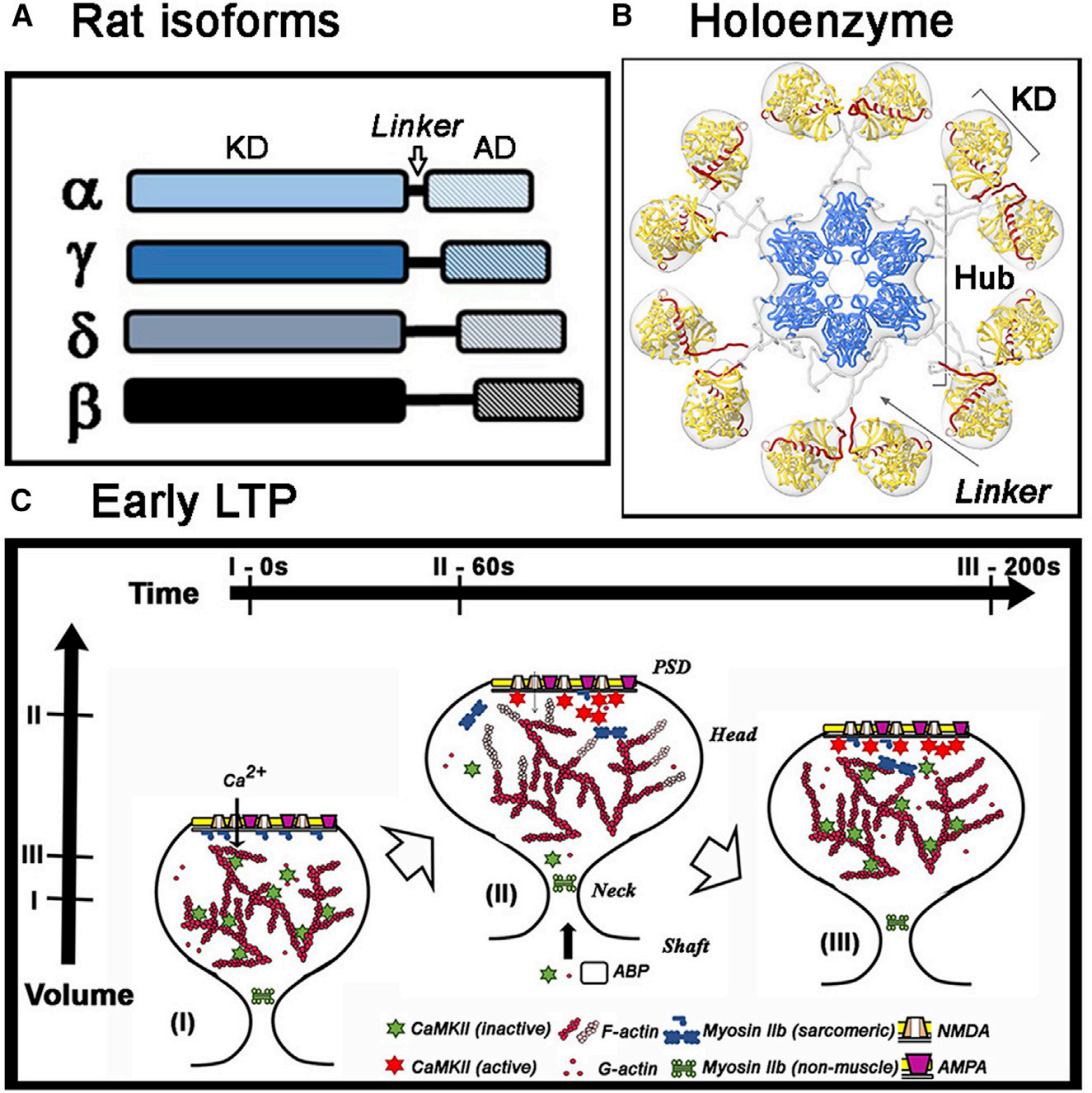
For a Figure360 author presentation of Fig. 1, see the figure legend at https://doi.org/10.1016/j.bpj.2018.11.006. (*A*) Rat isoforms. Subunits are distinguished by the length and composition of their linkers between the KD and AD. (*B*) Holoenzyme; Dodecamer with stacked hexamer rings. Linker extensions regulate kinase co-operativity possibly by controlling access of calcium-calmodulin to its R (regulatory segment (*red*))-binding motif. The dominant extended form of the autoinhibited CaMKII holoenzyme visualized by cryoelectron tomography of the rat *α*-isoform is shown (with permission from (20)). (*C*) Stimulus-dependent remodeling of the spine cytoskeleton. Spine morphology is shown in its initial (*i*) maximally expanded (*ii*) and stable end states (*iii*) after synaptic stimulation. (*i*) to (*ii*): transient (subsecond) calcium influx triggers calmodulin-mediated CaMKII activation, dissociation from F-actin (*red*), and sequestration to the PSD. CaMKII kinase activity orchestrates actin polymerization (F-actin*; pink*) to expand the cytoskeleton. ABP, actin binding protein. Compaction may be powered by PSD-localized myosin miniature-filament formation mediated by MLCK kinase activation. (*ii*) to (*iii*): as intracellular calcium returns to basal levels, compaction of the expanded cytoskeleton by myosin is completed and stabilized by the attachment of CaMKII that has entered from the shaft. Horizontal bars denote time stamps for the states, whereas the vertical bars mark relative spine head volumes. Figure360: An Author Presentation of Fig. 1

The actin-binding properties of the rat isoforms have been the most studied (Fig. 1 *A*). The variable intervening linkers in the rat CaMKII *β*-isoform (*β*_rat_; residues 316–405) (11)) between the conserved canonical kinase domain (KD) and association domains (ADs) are an actin-binding determinant because affinity scales with linker length as reported by pull-down assays and electron microscopy (EM) of the sedimented bundles (7, 12, 13). The 90-residue *β*_Rat_ linker, named actin-binding domain (7), has an established role in actin binding, but isoforms with shorter linkers also bind actin (*α*_Rat_: about 25 residues (14)) and form F-actin networks (*γ*_Rat_: about 40 residues), with *γ*_Rat_ reported to bundle F-actin differently than *β*_Rat_ (15). It is not known whether there is a common binding surface for both globular actin (G-actin) and F-actin or whether the architecture of CaMKII-actin networks fits the characterization of CaMKII as an “actin-bundling” protein (7).

Holoenzyme architecture is conserved between species, with the KDs arrayed around a central AD hub (Fig. 1 *B*). Autoinhibited states that bind actin have an incomplete ATP binding site blocked by a pseudo-substrate regulatory sequence (*β*_Rat_275–315 segmented into R_1_275–291, R_2_292–298, and R_3_308–315) (16). The association of *β*_rat_ to G-actin (2.4 *μ*M affinity) (13) with neuronal F-actin cytoskeleton (17) is abolished by calcium-calmodulin, noted to be within 5 s in the latter study. G-actin occupancy is stoichiometric with the number of subunits in the *β*_rat_ holoenzyme (13). Calcium-calmodulin binds to an R_2_ IQ-10 recognition motif to displace the regulatory sequence from the substrate-binding cleft, enabling its capture and R_1_ T287 transphosphorylation by an adjacent subunit whose regulatory sequence has also been displaced by calcium-calmodulin. This capture process (i.e., subunit capture) is essential for kinase activation (18, 19). Linker length is also known to regulate kinase cooperativity (18). Thus, the linkers could control access of calcium-calmodulin to its binding motif in autoinhibited holoenzymes as well as in multivalent holoenzyme interactions with F-actin by limiting the extension of KDs out from the central hub. Three-dimensional cryoelectron tomography has reported three distinct autoinhibited *α*_Rat_ holoenzyme populations: a dominant (>60%) population with extended KDs, and compact monomer (<30%), and paired dimer (<20%) populations (20). Accordingly, we used the structurally characterized *C. elegans* CaMKII “d”-isoform (*d*_*C. elegans*_) (18) and the human CaMKII*β*-isoform (*β*_Hum_) (21) that have threefold shorter and longer linkers than *β*_Rat_, respectively, to vary linker length over a large range to evaluate the role of linker length as a molecular determinant for CaMKII network formation and response to calcium-calmodulin.

Early LTP after calcium influx upon synaptic stimulation is defined by localized changes in the size of the stimulated spine. Calcium transients (<0.1 s) triggered by the photorelease of glutamate (22) cause expansion (3× basal volume) followed by compaction to a final size (1.5×) within 3 min (Fig. 1 *C*). The volume increase has been described as a “synaptic tag” (23, 24) because it is maintained for >1 h by as yet unknown mechanisms that direct the insertion of additional N-methyl-D-aspartate (NMDA)/α-amino-3-hydroxy-5-methyl-4-isoxazole propionic acid (AMPA) receptors at the postsynaptic membrane (PSD) at the synapse.

Single-particle tracking (SPT) of CaMKII reported three kinetic subpopulations in spines, with the major immobile subpopulation attached to the actin cytoskeleton (5, 25). Actin treadmilling, concomitantly monitored by photoactivation of PaGFP-actin with glutamate photorelease, revealed dynamic (<1 min time constant) and stable, enlarged (∼17 min time constant) F-actin pools. CaMKII inhibitors blocked formation of the latter pool (26). Thus, CaMKII determines the stability of the spine cytoskeleton. A stable cytoskeleton is essential to distinguish the volume of the stimulated spine from adjacent spines that do not change size. Thus, a central architectural role of CaMKII-actin networks, investigated in this study, could be the suppression of spontaneous volume fluctuations caused by osmotic imbalance and myosin motor forces that degrade the stimulus-induced volume difference between stimulated and nonstimulated spines. Reconciliation of the measured, weak actin-binding affinity with the long-term (>1 h) stability of CaMKII-actin networks in vivo needed to measure this difference is an important related issue.

There is evidence that the initial expansion during early LTP is due to actin polymerization, to which increased barbed ends because of F-actin severing proteins such as cofilin (23), F-actin fragmentation by membrane-associated myosin motors (27), and G-actin dissociation from CaMKII by calcium-calmodulin (15) could all contribute. In contrast, factors responsible for the subsequent compaction remain to be identified. An attractive hypothesis, formalized in a recent model (28), is that compaction is driven by the sarcomeric myosin-II isoform MyH7B tethered to PSD-associated SynGap/PSD-95/NMDA receptor complexes (29), consistent with the enlargement of spine size upon MyH7B knockdown (30). Myosin-IIb also regulates PSD size (31, 32) and could be an alternative motor. Another possibility is that osmotic depletion forces alone are sufficient for compaction of the newly polymerized F-actin. We sought to discriminate among these possibilities as well as understand how calcium-calmodulin dissociation of the CaMKII holoenzymes from F-actin is coupled to the cytoskeletal response to physical forces.

Responses of actin networks to crowding agents and myosin motors have been characterized previously (33, 34, 35, 36, 37). Here, we exploited these in vitro assays to assess whether motor and/or osmotic forces may drive the compaction phase of early LTP. We developed quantitative measures of network architecture and combined network analysis with single-molecule measurements of CaMKII association with G- and F-actin to understand how calcium-calmodulin-triggered dissociation of CaMKII is coupled to architectural dynamics the transition from a stable to dynamic network. We report that CaMKII cross-linked actin networks have sufficient mechanical resilience to maintain a rigid, stable architecture but disassemble rapidly in response to a calcium-calmodulin pulse. The disassembled F-actin is remodeled by myosin motor forces with kinetics consistent with the reported changes in stimulated spine volume. We document that, in contrast to kinase cooperativity, network architectural dynamics are relatively insensitive to linker length. Our results have general relevance for CaMKII-guided cytoskeletal modeling in other organs and tissues populated by non-neuronal isoforms.

## Materials and Methods

### Materials

Actin antibody specific to the C-terminus of *α*-actin, no. A5060 (rabbit), was purchased from Sigma (St. Louis, MO). Monoclonal GFP antibody no. 1814460 (mouse) was purchased from Roche (Basel, Switzerland), and the GFP protein, no. 8365-1, was from Clontech (Mountain View, CA). A 21-residue peptide, tatCN21, homologous to the NMDA type GluN2B receptor subunit (38), was a gift from Dr. Ulli Bayer. Polyethylene glycol (PEG) (MW 3KD) was from Sigma.

Buffer stocks for fluorescence assays were AB^−^ (25 mM imidazole-HCl, 25 mM KCl, 1 mM EGTA, and 4 mM MgCl_2_ (pH 7.4)) and AB^+^ (as AB^−^ but with additional 2 mM ATP). Ca^2+^/AB^−^ and Ca^2+^/AB^+^ contained 0.2 mM CaCl_2_ in place of 1 mM EGTA. Calmodulin, purified as detailed below, was used at 10 *μ*M final concentration unless noted otherwise. Immediately before the experiments, an oxygen scavenger system was added to 1-mL aliquots of the relevant degassed buffers to reduce photobleaching. This comprised 20 mM dithiothreitol, 0.2 mg/mL glucose oxidase, 0.5 mg/mL catalase, 3 mg/mL glucose, and 0.5 mg/mL bovine serum albumin (final concentrations). PEG solutions were made in AB^−^ buffer with GOC.

**Figure 2:**
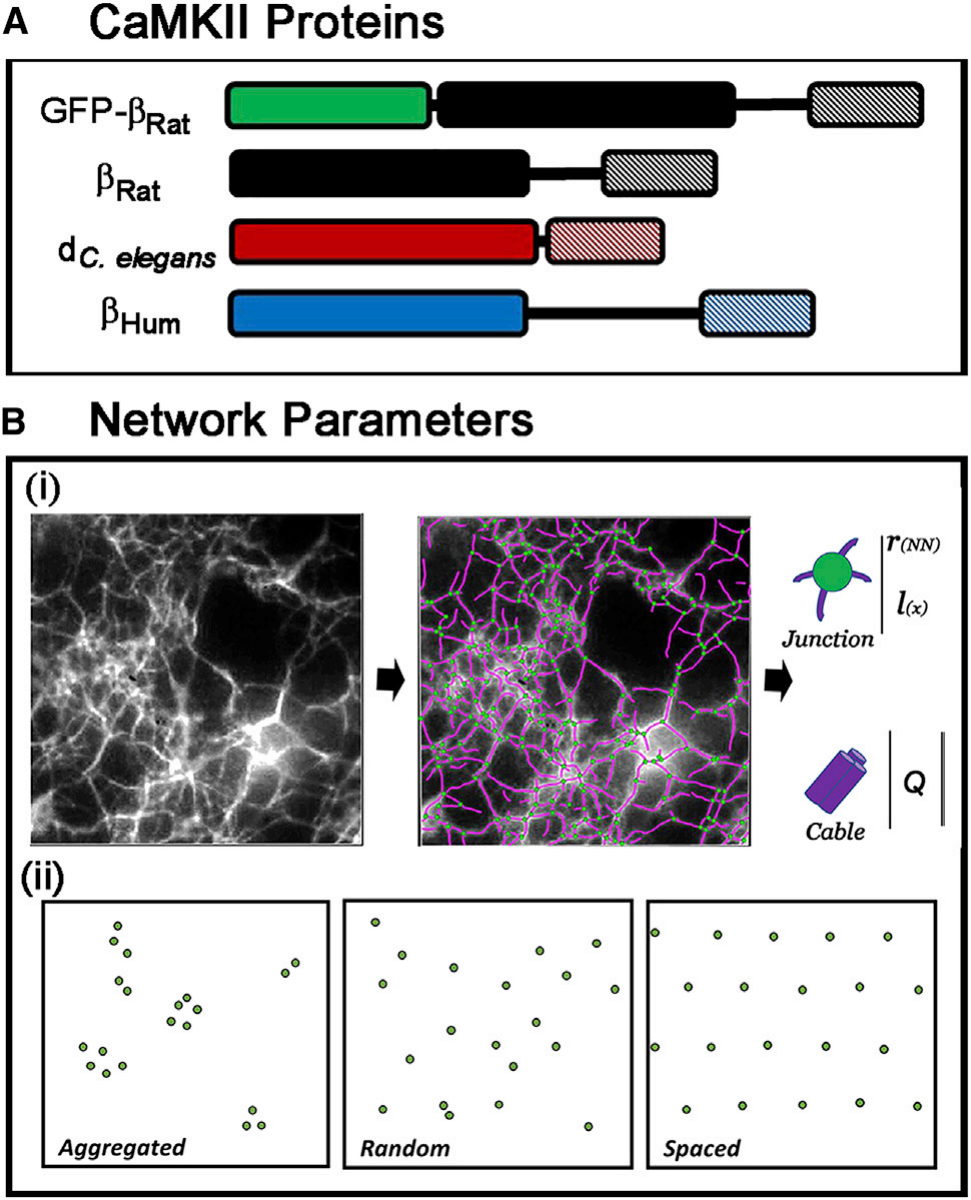
(*A*) CaMKII proteins used in the study. The three proteins (*β*_rat_ = rat *β*-isoform, *β*_Hum_ = human *β*-isoform, *d*_*C. elegans*_ = *C. elegans* splice variant d) were selected based on differences in linker lengths and phylogenetic spread. Single-molecule measurements utilized the GFP-tagged fusion of the rat *β-*isoform. (*B*) Network parameters. (*i*) SOAX representation of an F-actin network in 15% PEG showing snakes (*purple filaments*) and junctions (*green spots*) superimposed on the image. The parameters extracted from the SOAX were the junction density, interjunction separation (*l*_*(x)*_), and cable intensity (*Q*). The nearest-neighbor distance (*r*_*ΝΝ*_) was computed from junction density and separation. (*ii*) Network architectures. Random network flanked by examples of aggregated and spaced networks are shown.

The CaMKII proteins studied (Fig. 2 *A*) were chosen based on their interdomain linker properties and phylogenetic spread (Supporting Materials and Methods, Section A). The structurally characterized *C. elegans* CaMKII-d-isoform (*d*_*C. elegans*_) (18) has a threefold shorter linker than *β*_Rat_, which in turn has a threefold shorter linker than the human CaMKII*β*-isoform (*β*_Hum_) (21). The *C. elegans* splice variants were confined to a branch within the phylogenetic trees (monophyletic) for both the KD and linker domains in contrast to the rat and human CaMKII isoforms that were distributed over the trees. The linker domains of the three proteins shared sequence homology over two small segments: a previously identified core fragment (*β*_Rat 342_KSLNNKKAD_351_ (11)) and another serine/threonine-rich 20-residue peptide at the *β*_Rat_ linker/AD interface (Fig. S1). The linkers were disordered, as assessed by DisoPred (39), so that linker compliance was proportional to linker length (Fig. S2).

The rat CaMKII*β*-isoform (*β*_Rat_) with an N-terminal endogenously biotinylated sequence (Avi tag) followed by a nine-residue flexible linker (3× Gly-Ala-Ser) was synthesized and cloned into plasmid pSMT3 (GENEWIZ). Plasmid pSMT3 contains a SUMO protease cleavage site downstream of an N-terminal His-6 tag. The GFP-tagged rat CaMKIi*β* (GFP-*β*_Rat_) has been described previously (14). The structurally and biochemically characterized *C. elegans* splice variant d (*d*_*C. elegans*_) (18) and the human *β*-isoform (21) were the two other CaMKII holoenzymes studied. Their subunits are expressed with N-terminal His-6 tags that are cleaved by prescission and SUMO protease, respectively. Further details on protein expression, purification, and negative-stain EM are in Supporting Materials and Methods, Section A.

### Epifluorescence and total internal reflection fluorescence light microscopy

Epifluorescence microscopy assays studied rhodamine (Rh)-phalloidin (Ph) stabilized F-actin gels and their motile response to crowding and motor forces. The assays used a Zeiss Axioskop 40 upright fluorescence microscope (Oberkochen, Germany) (40) equipped with a 100-W mercury arc lamp, rhodamine filter set (Ex:525AF45, dichroic:560DRLP, Em:595AF60) (Omega Optical, Brattleboro, VT), 100× Plan Neofluar (1.3 numerical aperture objective), and PTI IC300 image-intensified charge-coupled device camera (Horiba, Kyoto, Japan). Camera magnification was 78 or 119 nm/pixel (selected using a 1.5× magnification lens). Video records were captured using a Euresys Picolo frame grabber card (Multipix Imaging, Petersfield, Hampshire, UK) and recorded onto a computer hard disk. Defined depth chambers were used to estimate GFP-*β*_rat_ concentrations in extracts, with known GFP solutions as the calibration standard. Dual-color, single-molecule total internal reflection fluorescence (TIRF) imaging studies were conducted using a Nikon Eclipse TE 2000U inverted microscope (Tokyo, Japan) as described previously (14) but with modifications.

### Image analysis

#### Single particles

SPT was performed with GMimPro and further analyzed with Motility (41) software. Kymograph and colocalization analysis used ImageJ plugins. TIRF sample preparation, instrument modifications, and image analysis details are in Supporting Materials and Methods, Section B.

#### Networks

The SOAX program, based on Stretching Open Active Contours (42), was used for automated representation of the light microscopy (LM) imaged networks (sampling distance, *c* = 8 pixels (0.625 *μ*m)). SOAX gave a map of the coordinates of filament crossover points (i.e., junctions) and outlines of the connecting cables (i.e., snakes) superimposed on the original image (Video S1). Snakes included both single filaments and bundles that could not be distinguished in the diffraction-limited images. SOAX parameters were iteratively optimized for maximal superposition of the map with the imaged network. Manual editing eliminated spurious snakes and fused neighboring junctions <1.2*c* apart. Junction centroids and all interjunction distances, *l*_*x*_, were determined from the map. Nearest-neighbor distances, *r*_*NN*_, were then obtained from the table of *l*_*x*_ values using a custom script in R 3.3 (https://www.r-project.org/). The average near-neighbor distance, *R*_*NN*_, was computed from the observed number, *N*, of *r*_*NN*_ values, expressed in *μ*m:

(1) $$R_{NN}=\;\sum r_{NN}/N\text{.}$$

Video S1. SOAX Representation of an F-Actin Network, Related to Fig. 2 *A*

The mean snake intensity, *Q*, was determined in detector counts per image pixel area. The skewness of the intensity distribution, *S* (0 for symmetric distribution with mean = mode), was computed in Microsoft Excel. Mean snake curvature, *K*, measured in radians per micrometers was computed in SOAX from the tangent differences, *Δ*T, at user-defined sampling distances, *Δ*s, along the snake:

(2) K=(1n)∑s=1nΔTΔs.

Calculation of the persistence length $\left(l_{p}\right)$ from *K* assumed a worm-like chain:

(3) P(K)=2lpcπ(exp(−lpcK2/2)),

where *P*(*K*) is the probability density distribution for *K* (further details are in (43)).

Our quantitative analysis of network type was based mainly on *Q* and *R*_*NN*_ (Fig. 2 *B*), with *l*_*p*_ used as a qualitative check only because it was systematically underestimated (Supporting Materials and Methods).

For networks compacted by myosin motors, the fluorescence foci comprising conglomerated F-actin were identified from binarized images by setting area (>50 *μ*m^2^) and intensity thresholds using the particle analysis function in ImageJ version 1.5 (https://imagej.nih.gov). Foci centroid positions, interfoci distances (*l*_*x*_), and nearest-neighbor distances (*r*_*NN*_) were computed as above for the snake junctions to obtain the integrated intensity, $\sum Q_{foci\;}$:

(4) $$\;\sum Q_{foci\;\;}=\sum_{foci=1}^{n}A_{foci}Q_{foci}\text{,}$$

where *A*_*foci*_ (pixel^2^) and *Q*_*foci*_ counts per pixel are the area and mean intensity for each of *n* foci in an image field of view, using the binarized image to mask all foci located on the original image.

*R*_*NN*_ was used to characterize the network type by comparison against the expected value for a random (Poisson) distribution, *r*_*rand*_, at a given density of junctions, *σ*, per unit area. It has been shown that for a random distribution obtained when the position of any point is equiprobable and independent of all other points:

(5) $$r_{rand}=1/2\sqrt{\sigma }\text{.}$$

The limiting extreme divergences from the random distribution are an aggregated distribution, in which points coalesce and *R*_*NN*_ approaches zero, and an optimally spaced distribution on a hexagonal point spacing (Fig. 2 *B*), where $R_{NN}=\;1.0796/\sqrt{\rho }$ (44). Here, we define a randomness index, *R* = *R*_*NN*_/*r*_*rand*_, for our network architectures. *R* will vary from ∼0 for the aggregated case to unity for a random (noninteracting) network and up to 2.16 for the evenly spaced hexagonal lattice.

## Results

### Multivalent linkages by single holoenzymes connect CaMKII-actin networks

**Figure 3:**
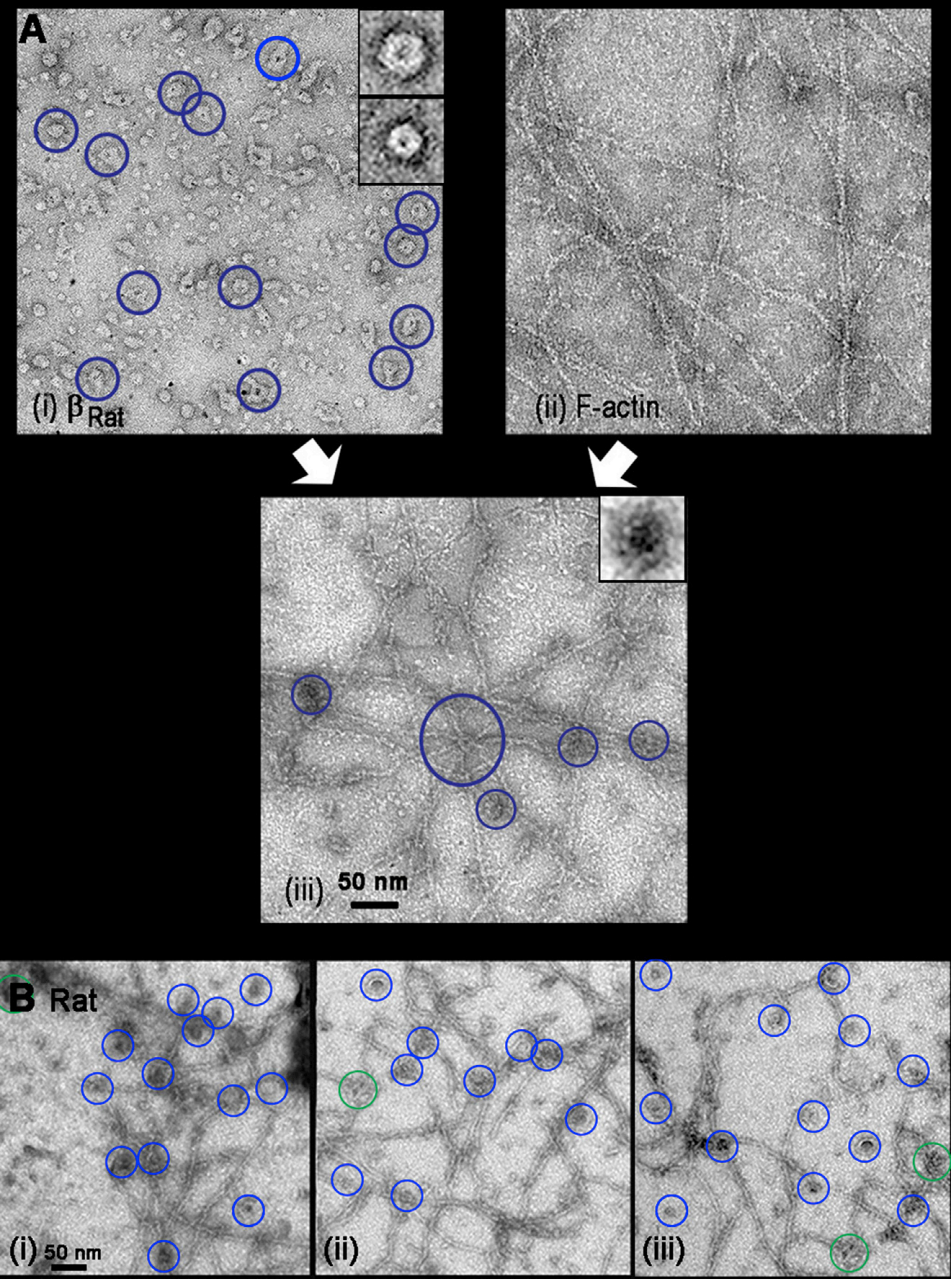
Architecture of *β*_Rat_-actin networks. (*A*) (*i*) *β*_Rat_ and (*ii*) F-actin solutions form (*iii*) networks when mixed. Some holoenzymes are identified (*blue circles*). Boxes (45 nm) show image averages of the dominant subpopulations (*top*: holoenzymes (*n* = 21; 25.8 ± 0.8 nm); *bottom*: proteolyzed hubs (*n* = 15; 18.0 ± 0.3 nm)). (*iii*) Multiple (six) F-actin filaments joined by one holoenzyme (*center*; *large blue circle*). Junctions with two or three filaments formed by single holoenzymes (*blue circles*) are common. The box (45 nm) shows the image average of junction-localized *β*_Rat_ (*n* = 30; 24.0 ± 2.6 nm). (*B*) (*i*–*iii*) *β*_Rat_-actin networks. Junctions have one (*blue circles*) or two (*green circles*) holoenzymes.

Negative-stain EM was used to resolve the interfilament F-actin connections by *β*_Rat_ holoenzymes in networks formed in the presence of physiological substoichiometric *β*_Rat_/G-actin molar ratios (0.3) (Fig. 3 *A*). The networks were a composite of two structural elements (i.e., crossover junctions and bundles) in addition to free single filaments. The *β*_Rat_ holoenzymes localized preferentially at junctions and along bundles when mixed with synthetic F-actin. Multiple filaments were connected dominantly by single holoenzymes at junctions rather than aggregates. Proteolysis of the unstructured linker (Fig. S3 *A*) resulted in smaller AD hubs and cleaved KD complexes in addition to intact holoenzymes. The hubs did not localize at junctions.

**Figure 4:**
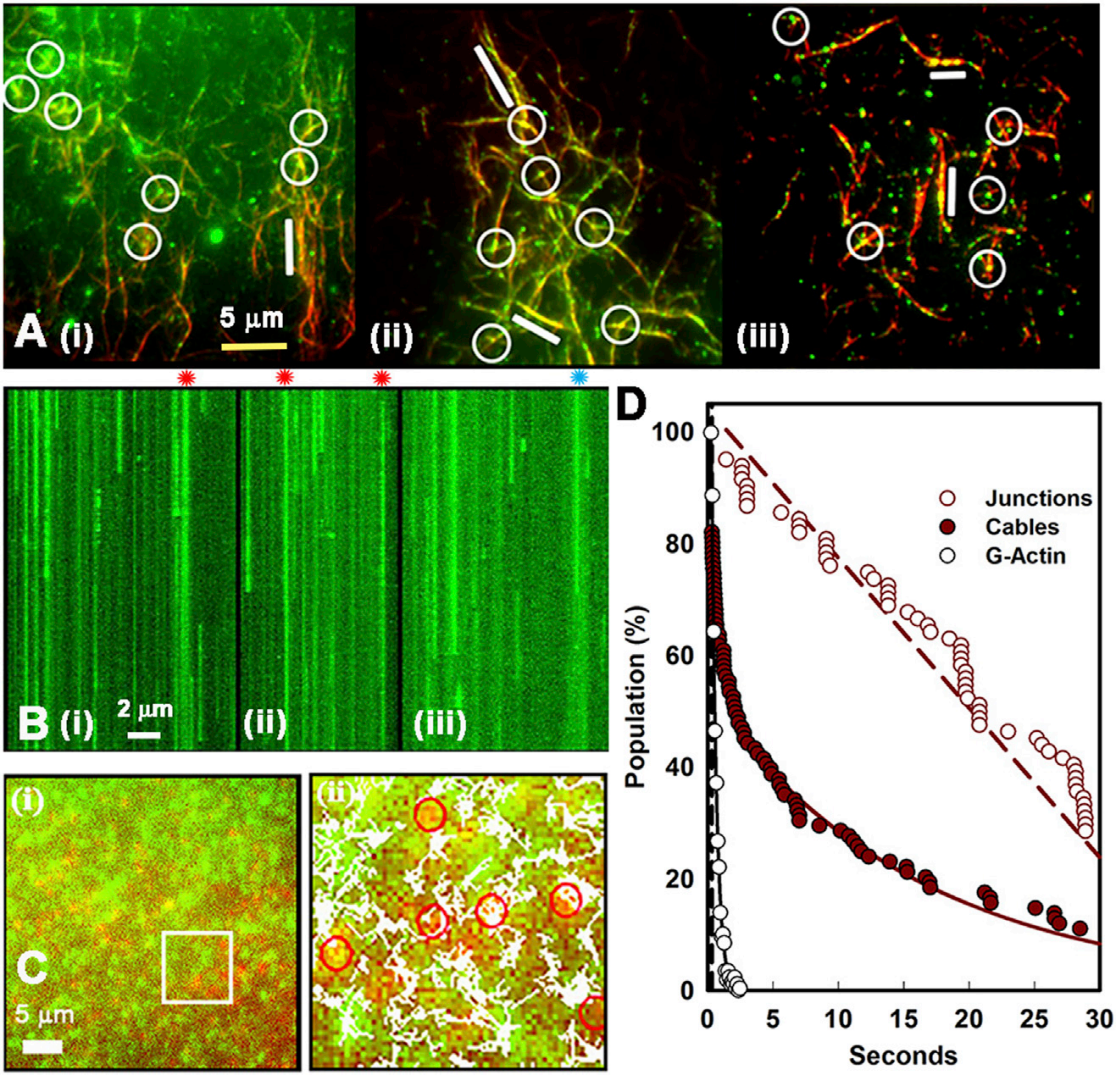
Single-molecule binding to F- and G-actin. (*A*) (*i*–*iii*) Averaged dual color TIRF of GFP-*β*_Rat_ holoenzymes (*green spots*) bound to Rh-Ph F-actin (*red*) filament aggregates (*R* = 0.58; *S* = 1.05). 20 frames/s video frame rates were used. AB^−^/GOC buffer is shown. 100 frame averages are shown. Circles (*white*) mark GFP-*β*_Rat_; bars (*white*) mark filament stretches decorated continuously with GFP-*β*_Rat_. (*B*) (*i*–*iii*) Kymographs of green fluorescent spots associated with actin filaments. Asterisks (*red*) mark spots at junctions. The duration (abscissa) of each kymograph is 20 s (400 frames). Length scale (*white*) represents 2 *μ*m. Bound fraction (dwell time > 6 frames (0.3 s)) is 0.82 ± 0.11. (*C*) (*i*) GFP-*β*_rat_ (*green*) molecules in the evanescent field adjacent to a surface layer of antibody immobilized Cy3 G-actin (*red*) molecules. (*ii*) Particle tracks superimposed on a snapshot of part of the image field (*box area* in (*i*)). (*D*) Values for *k*_*off*_ were determined from single- or double-exponential best fits (*lines*) to dwell-time distributions. Subpopulations localized at junctions (*open red circles*; *k*_*off*_ = 0.04 s^−1^) versus cables (*solid red circles*; rate = 0.43(exp (−1.2) + 0.56(exp (−0.06) s^−1^) are shown. The distribution of GFP-*β*_rat_ track dwell times over the immobilized Cy3 G-actin fits single-exponential decay (*white*; k = 2.55s^−1^; *R* > 0.99) is shown. The vertical dashed line marks six-frame (0.3 s) “bound” spot threshold.

We set up single-molecule total internal reflection fluorescence microscopy (TIRFM) assays to test whether *β*_rat_ holoenzymes attached more strongly at network junctions (i.e., “filament intersections”) consistent with the multiple attachments to actin filaments seen by EM. The GFP-*β*_rat_ construct used has been extensively characterized in several laboratories, including ours (6, 12, 14). The GFP-*β*_rat_ holoenzymes, once dissociated from binding targets, rapidly (<5 video frames (0.1 s)) diffuse out of the evanescent field, as detailed in an earlier publication (14). Henceforth, we use the term “cables” for both single filaments and bundles because filaments within a bundle cannot be resolved by LM. Network architecture was characterized based on two parameters: cable intensity (reported by the normalized pixel intensity (*Q* value) that increases with the number of bundled filaments for the diffraction-limited case) and nearest-neighbor junction distance *R*_*NN*_ compared to that expected for a random network, *r*_*rand*_. The mean and the skewness, *S*, of the *Q* distribution gave a measure of bundling. The randomness parameter *R* computed from the ratio (*R*_*NN*_/*r*_*rand*_) reported the arrangement of the filament junctions. Individual GFP-*β*_rat_ holoenzymes (10–50 nM concentration) appeared as diffraction-limited spots, which decorated the immobilized synthetic Rh-Ph F-actin filaments (Fig. 4 *A*; Video S2). We imaged areas enriched in F-actin bundles and junctions. The GFP-*β*_rat_ spots localized preferentially at junctions and along the brighter cables. Kymographs were generated to analyze the single-particle dwell-time distribution of GFP-*β*_rat_ holoenzymes that were localized along the F-actin (Fig. 4 *B*).

**Figure 5:**
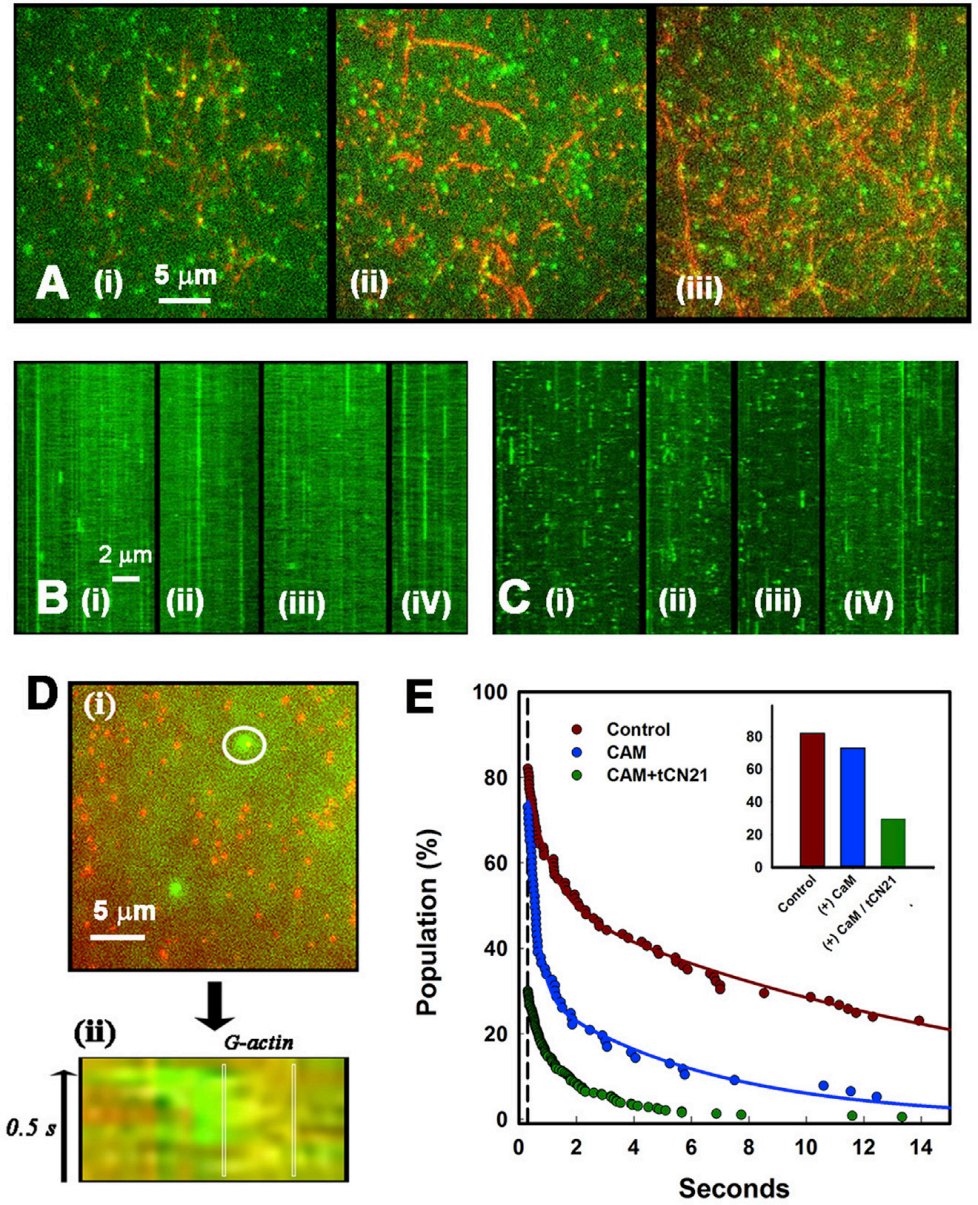
(*A*) Dual-color TIRF of GFP-*β*_Rat_ holoenzymes (*green spots*) associated with F-actin in the presence of (*i* and *ii*) calcium-calmodulin and (*iii*) calcium-calmodulin with tCN21 peptide; tow frame averages. Kymographs (20-s duration) of GFP-*β*_Rat_ spots associated with F-actin in the presence of calcium-calmodulin ((*B*) (*i*–*iv*)) and (*C*) (*i*–*iv*)) calcium-calmodulin with tCN21 peptide. (*D*) (*i*) A TIRF image of GFP-*β*_Rat_ molecules over a surface of immobilized Cy3 G-actin molecules. Concentrations and experimental conditions as in Fig. 4*C* but in the presence of calcium-calmodulin are shown. (*ii*) The kymograph (*circle* in (*ii*); 3-*μ*m diameter) shows transient interaction of GFP-*β*rat particle with a Cy3-actin molecule (demarcated). (*E*) Distributions of bound-control (*red*), calcium-calmodulin (*blue*; *k*_*off*_ = 0.78 (exp (−3.1) + 0.22(exp (−1.4) s^−1^), and calcium-calmodulin (+tCN21) (*green*; *k*_*off*_ = 1.0 s^−1^) treated populations above the six-frame (0.3 s) threshold. The inset shows fractions of [(bound spots (>6 frames threshold))/(Total spots)] − 0.82 ± 0.11 (control), 0.73 ± 0.09 (calcium-calmodulin), and 0.30 ± 0.01 (calcium-calmodulin + tCN21).

Video S2. TIRFM of GFP-*β*_rat_ Association/Dissociation with F-Actin ((+/−) Calcium-Calmodulin), Related to Fig. 4 *Aii* and Fig. 5 *Aii*

We compared the F-actin GFP-*β*_rat_ dwell-time distributions with those obtained with Cy3-labeled G-actin because GFP-*β*_rat_ attachments with G-actin would only be monovalent in contrast to F-actin. The Cy3-actin was immobilized as a surface layer on actin antibody-coated glass. GFP-*β*_rat_ formed a dense layer associated with the immobilized G-actin spots when perfused in (Fig. 4 *C*). The SPT dwell-time distribution was well fit by a single exponential, with off rate *k*_*off*_ = 2.55 s^−1^(n > 1200). This value provided an estimate of the monovalent *k*_*off*_. In contrast, analysis of the kymographs (Fig. 4 *D*) for the GFP-*β*_rat_ associating with the F-actin network revealed a long-tailed multiexponential decay. Junction-localized CaMKII had substantially longer dwell times (slower *k*_*off*_) than the rest of the population, implying that the steady-state observation of preferential CaMKII localization at junctions was due to slower dissociation. These results are most simply explained by the idea that the rotationally symmetric holoenzyme forms multivalent attachments with multiple filaments at network junctions. Detachment from all filaments, which is required for dissociation from a junction, should take longer than detachment from G-actin. In the case of single filaments, single attachments will constitute the dominant fraction. In cables with two or more filaments, higher-valency attachments will also be common. The mean GFP-*β*_rat_ spot intensity measured at actin-filament junctions was not significantly greater than the average intensity of spots localized at other points along F-actin, consistent with the EM evidence that junctions are predominantly formed by single holoenzymes.

### Single-molecule assays of dissociation by calcium-calmodulin

The rapid dissociation of activated CaMKII from the actin cytoskeleton for phosphorylation of PSD targets and G-actin for stimulating polymerization is central to models, advocating a primary role for CaMKII in the LTP spine volume change. Therefore, we measured the GFP-*β*_rat_ dissociation from both F-actin and G-actin triggered by calcium-calmodulin. When a mixture of calmodulin/GFP-*β*_rat_ (in Ca^2+^⋅AB^−^ buffer) was perfused onto the F-actin immobilized in the flow cell, GFP-*β*_rat_ decoration at both cables and junctions was substantially reduced within the time taken to complete the buffer exchange (∼20 s) (Fig. 5, *Ai* and *Aii*). This result implies that calcium-calmodulin activates and releases CaMKII rapidly enough to orchestrate the volume increase of stimulated spines. A residual GFP-*β*_rat_ fraction remained bound to F-actin. This fraction was unaffected by subsequent perfusion of calmodulin/GFP-*β*_rat_ with ATP (Ca^2+^ ⋅ AB^+^ buffer). We then used the peptide tCN21, an analog of the NMDA receptor, to explore whether the receptor interaction would reduce the residual bound fraction (Fig. 5 *Aiii*; Video S2). This fraction was indeed reduced when tCN21 was perfused with calmodulin/GFP-*β*_rat_ (Ca^2+^ ⋅ AB^−^ buffer). Threefold higher GFP-*β*_rat_ concentrations in the mixture relative to those used in the absence of tCN21 were necessary to obtain dwell-time distributions with acceptable statistics of spots bound longer than the five-frame threshold. This concentration difference is evident from comparison of the kymographs obtained for GFP-*β*_rat_-spot dwell times in the absence (Fig. 5 *B*) and presence of tCN21 (Fig. 5 *C*).

Similarly, there was a dramatic decrease relative to control experiments (Fig. 4 *C*) in the number of GFP-*β*_rat_ spots adjacent to immobilized Cy3-actin surface layers in the presence of calcium-calmodulin (Fig. 5 *Di*) with high background fluorescence due to distant particles outside the field. A few GFP-*β*_rat_ particles diffused in and out of the evanescent field with brief stationary episodes in proximity to G-actin spots as realized by kymograph analysis (*n* = 12). An example kymograph is shown (Fig. 5 *Dii*). The difference between Cy3-actin experiments with and without calcium-calmodulin is best appreciated from the video records (Video S3). Alternatively, anti-GFP antibody-coated glass coverslips immobilized GFP-*β*_rat_ molecules that remained visible as diffraction-limited spots when the extract was replaced with AB^−^. Multiple Cy3-actin molecules, as assessed by the *Q* value, colocalized with a dominant fraction of the GFP spots when Cy3-actin solutions were then perfused in. Perfusion of calmodulin (Ca^2+^ ⋅ AB^−^ buffer) triggered a large decrease in colocalization with dissociation kinetics that was again too rapid to be resolved with flow-cell exchange. About 20% of the colocalized Cy3-actin was also refractory to dissociation (Fig. S3, B and C).

Video S3. TIRFM of GFP-*β*_rat_ Association/Dissociation with G-Actin ((+/−) Calcium-Calmodulin), Related to Figs. 4 and 5

### Conservation of network architecture and calmodulin sensitivity

**Figure 6:**
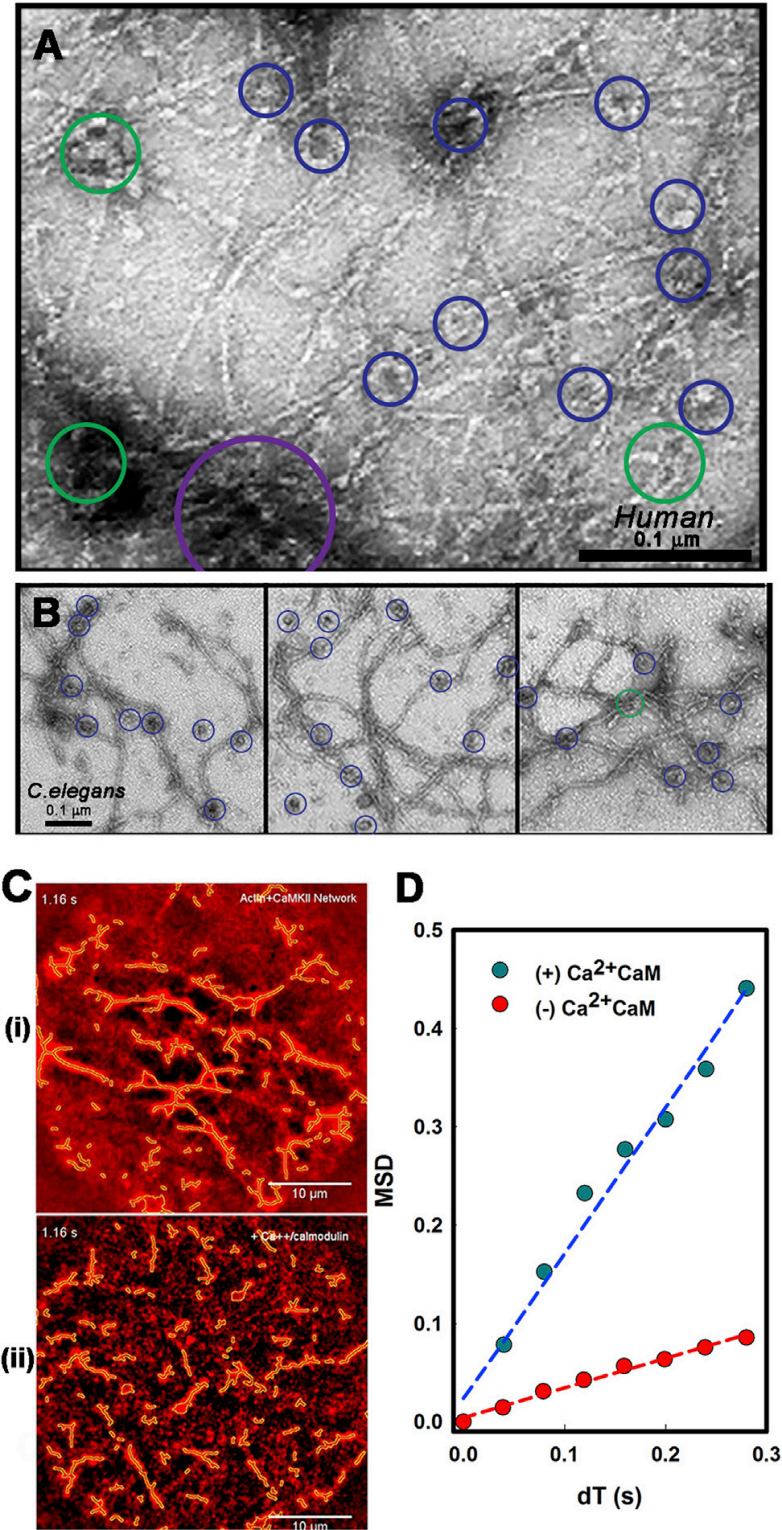
Negative-stain EM images of actin networks. (*A*) *β*_Hum_. Junctions with one (*blue*) or two (*green*), or holoenzymes are circled (holoenzyme/junction = 1.35 ± 0.6). The aggregate of multiple holoenzymes (*purple*) decorates a bundle. (*B*) *d*_*C. elegans*_-actin networks with junctions color coded as in (*A*). Scale bars, 0.1 *μ*m. (*C*). Video frames with filaments are outlined by the GMimPro tracking algorithm for centroid computation. (*i*) Rh-Ph biotinylated F-actin gel with *d*_*C. elegans*_ on streptavidin-coated glass after 30-min of incubation with *d*_*C. elegans*_ (*Q* = 13,030 counts/pixel; *R* = 0.75) (*ii*) Disassembled filaments <3 min after subsequent flow-in of 0.1 mM calcium/1-*μ*M calmodulin. (*D*) Mean-square deviation versus time interval (dT) plots for populations before (*red symbols*; *D*_*lat*_ = 0.06 *μ*m^2^/s) and after (*blue symbols*; *D*_*lat*_ = 0.37 *μ*m^2^/s) treatment with calcium-calmodulin.

We next investigated whether the design principle of a cross-linked network created from a multivalent attachment of single holoenzymes was preserved when linker length was varied. The formation of *β*_*Hum*_ (Fig. 6 *A*) and *d*_*C. elegans*_ (Fig. 6 *B*) actin networks demonstrated this was the case and that, furthermore, the association of CaMKII with F-actin was conserved in invertebrate as well as invertebrates. The large variation in linker length between these species (25 *d*_*C. elegans*_ and 200 *β*_hum_ residues) allowed us to expand on the relationship between linker length and actin binding deduced from the rat isoforms (Introduction). Bundles and junctions should have dominated the *β*_*Hum*_ network if linker compliance facilitated multivalent association as proposed (14), but this expectation was not met. Quantitative comparison of the networks formed by the three CaMKII species showed that the mean number of filaments at single holoenzyme junctions was similar (2.65 (*β*_*Rat*_), 2.5 (*d*_*C. elegans*_), and 2.79 (*β*_*Hum*_)) (Fig. S4 *A*). There was an increase in the fraction of *β*_*Hum*_ network junctions with two or more holoenzymes (0.4 versus 0.1 (*β*_*Rat,*_); 0.18 (*d*_*C. elegans*_)), possibly because the long, unstructured *β*_*Hum*_ linker may promote self-association. The *d*_*C. elegans*_ and *β*_hum_ networks had similar porosity to *β*_*Rat*_ at similar molar concentration. The *d*_*C. elegans*_ network was less dense than the *β*_*Rat*_ one based on the junction density, but this may simply reflect grid-to-grid variation.

Linker length could regulate calcium-calmodulin access as noted in Introduction. If so, networks formed by the short-linker *d*_*C. elegans*_ should resist disassembly by calcium-calmodulin. We used epifluorescence LM to test whether this was the case. The *d*_*C. elegans*_ molar concentration was increased threefold to form a network in which most cables were bundled. The network disassembled completely within 1 min when perfused with calcium-calmodulin as measured by increased thermal motions (Video S4). Filaments could be tracked for centroid computation in single video frames despite the background epifluorescence (Fig. 6 *C*). The mobility was documented by mean-square deviation versus dT plots before and after perfusion (Fig. 6 *D*) with the anisotropic diffusion of the disassembled filaments evident in the tracks.

Video S4. *C. elegans* CaMKII-Actin, Network ((+/−) Calcium-Calmodulin), Related to Fig. 6, *C* and *D*

In conclusion, the EM established that the nematode holoenzyme with short linkers forms networks almost as well as the longer linker vertebrate holoenzymes at physiological molar ratios based on the common design of multivalent junctions formed by one or two holoenzymes. The single-molecule TIRFM experiments demonstrated the activated CaMKII was released rapidly enough to phosphorylate targets relevant for cytoskeletal remodeling during early LTP. The LM tested that the short *d*_*C. elegans*_ linker does not impede access of calcium-calmodulin on the minute timescale, importantly documenting CaMKII-actin network disassembly as an additional mechanistic feature during early LTP with consequences for cytoskeletal remodeling as explored below.

### The bundling of actin filaments by crowding agents is blocked by CaMKII

The CaMKII-actin network disassembly triggered by calcium-calmodulin has physiological relevance only if the architecture of the assembled network is insensitive to perturbations experienced in live cells but malleable to such perturbations when disassembled. Motivated by this consideration, we examined the resilience of CaMKII-actin networks to molecular crowding and myosin motor activity in the absence and presence of calcium-calmodulin.

**Figure 7:**
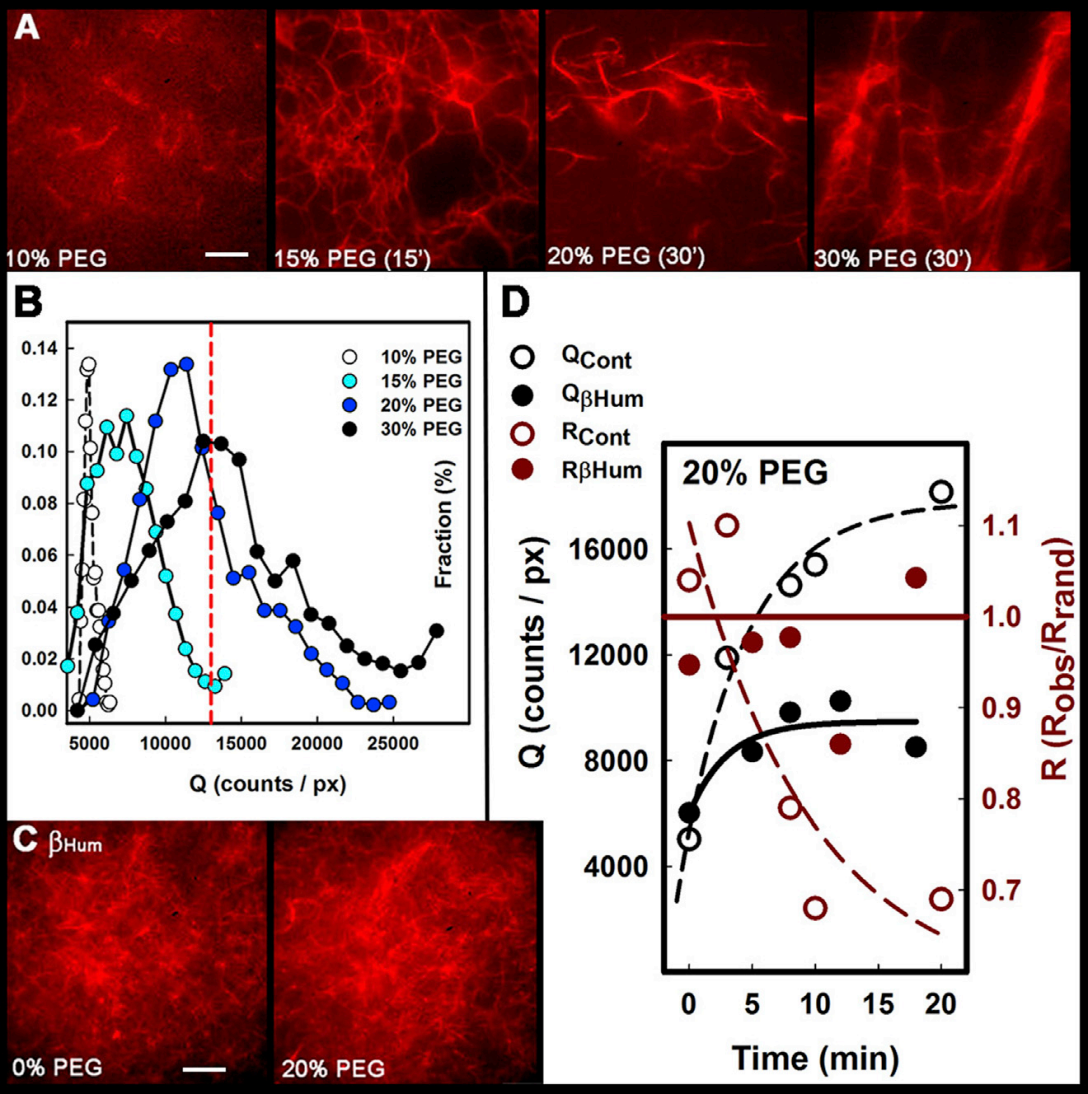
CaMKII counteracts macromolecular crowding. (*A*) Rh-Ph F-actin suspensions in AB^−^/GOC with 0% PEG, 15% PEG (15 min), 20% PEG (30 min), and 30% PEG (30 min). (*B*) Cable intensity (*Q*) distributions as a function of PEG concentration show shifts in modal values as bundling increases (*S* = 0.17 (0% PEG); 0.39 (10%PEG); 0.62 (15% PEG); 0.72 (20% PEG)). The dashed line (*red*) is the *Q* value for the *d*_*C. elegans*_ network (Fig. 6*Ci*). (*C*) Rh-Ph F-actin/*β*_Hum_ mixtures in 0% (*R* = 1.04; *Q* = 7810 counts/px (pixel)) and 20% PEG. Scale bars (*A* and *D*), 5 *μ*m. (*D*) Time course of bundling/network formation (20% PEG buffer; F-actin without (*open*) and with *β*_Hum_ (*solid*)) tracked by the increase in *Q* and decrease in *R*. Lines show single-exponential best fits.

PEG, an inert globular solute whose effects on F-actin organization have been studied previously (33), was used to study crowding (Fig. 7 *A*). The SOAX snake intensity (*Q*) distribution mapped the increase in bundling with PEG concentration (Fig. 7 *B*). The volume fraction is related to the difference between the solution and solvent viscosity (45). The viscosity of 20% PEG solution was estimated to be 8.6 centipoise (Fig. S4 B*i*), comparable to estimates for dendritic spine cytoplasm (46). The persistence length (*l*_*p*_) computed from snake curvature (*K*) increased (i.e., the filaments stiffened) from 4.5 *μ*m (10% PEG) to 10 *μ*m (20% PEG) with smaller increase at 30% PEG as the networks formed. The *K* distributions were fitted well by the worm-like chain model even though *l*_*p*_ was systematically underestimated (Fig. S4 B*ii*). Filament aggregation showed a sharp increase between 15 and 20% PEG concentration (Fig. 7, *A* and *B*). However, CaMKII-actin networks formed with *β*_Hum_ (or *β*_Rat_) in the absence of calcium-calmodulin, but in the presence or absence of 20% PEG, they did not aggregate (Fig. 7 *C*). 20% PEG caused F-actin alone and F-actin mixed with *β*_Hum_ and calcium-calmodulin to aggregate in ∼20 min as tracked by changes in *Q* and *R*. In contrast, 20% PEG mixed with F-actin and *β*_Hum_ in the absence of calcium-calmodulin showed a substantially smaller increase in *Q* and no significant change in *R* (Fig. 7 *D*).

We conclude that aggregation occurs when the network is disassembled by calcium-calmodulin, but the kinetics is too slow (>20 min) to account for the compaction phase recorded during early LTP. Importantly, the CaMKII-actin networks are not deformed by osmolytes at solution viscosities compared to those obtained in live cells. The networks would therefore maintain spine morphology under basal conditions.

### Remodeling by myosin motors requires network disassembly

**Figure 8:**
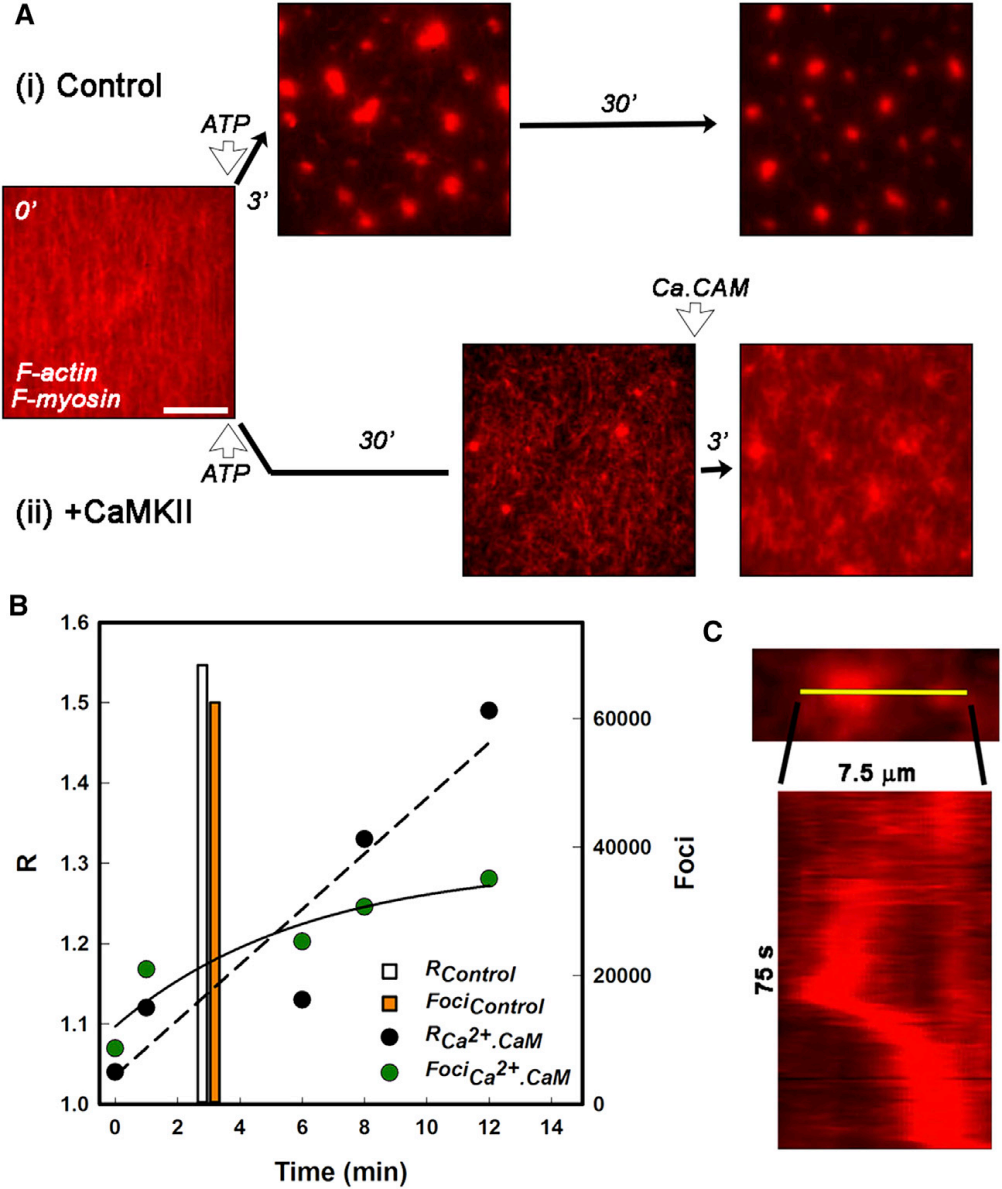
Long-range alignment by myosin motor myosin filaments. (*A*) Rh-Ph biotinylated F-actin tethered to streptavidin-coated glass coverslips incubated with myosin filaments. Compaction after perfusion of AB^+^/GOC (2 mM ATP) in the absence (*i*) and presence of (*ii*) *d*_*C. elegans*_. After perfusion of calcium buffer Ca-AB^+^/GOC with 1 *μ*M calmodulin, the CaMKII cross-linked network changes to resemble the compacted control network. Scale bars (*white*), 5 *μ*m. (*B*) Time course of network remodeling after calcium-calmodulin perfusion tracked as an increase in *R* (*black*) and $\sum Q_{foci\;}$(foci; *green*) compared to the time for foci formation (*R*; *white*), $\sum Q_{foci\;}$(*orange*) in the absence of CaMKII. Linear (*dashed line*) and exponential (*continuous curve*) best fits to *R* and *Q*, respectively, are shown. (*C*) A kymograph of the approach and coalescence trajectories of two foci (from a central section of the image field (Video S5; with calcium-calmodulin) along the yellow line.

Next, we tested the effect of the mechanical resilience conferred by CaMKII to forces exerted on actin networks by myosin filaments. Surface-tethered, three-dimensional CaMKII-actin networks were created by adding biotinylated F-actin (1:10 biotin/G-actin labeling ratio) to a neutravidin-coated coverslip followed by perfusion of different mixtures of nonbiotinylated Rh-Ph F-actin, myosin miniature filaments, and *d*_*C. elegans*_ CaMKII in AB^−^. After 30 min of incubation, unbound material was washed away with AB^−^, and networks were viewed by epifluorescence microscopy. First, a control experiment, performed in absence of CaMKII, showed that ATP addition (AB^+^) triggered rapid compaction of the actin filaments as reported (36). Fluorescent F-actin foci coalesced (<3 min) and compacted until the force balance between myosin filaments at different foci caused them to become spaced and pulled the interconnecting F-actin cables taut. F-actin networks were then formed in the presence of CaMKII. Now, compaction was largely blocked over a 30-min period after the addition of ATP. Subsequent addition of calcium-calmodulin initiated rapid compaction that was >50% complete within 3 min (Fig. 8 *A*; Video S5). After 6 min, additional foci did not form, but the intensity of existing foci and the spacing between them increased. The *R* value increased linearly over a 12-min period (i.e., the foci spacing became more ordered with time), whereas the integrated foci intensity $\sum Q_{foci\;}$showed a single exponential increase to a limiting value (Fig. 8 *B*). Coalescence dynamics of two adjacent foci with brief episodes of rapid translocation over a 75-s period (0.1 *μ*m/s approach rate) are shown in an example kymograph (Fig. 8 *C*). The rate of coalescence was comparable to the reported control rate (36) indicating that any residual CaMKII-actin attachments do not impede the mechanics.

Video S5. Myosin Mmini-Filament Powered Compaction ((+/−) CaMKII, (+/−) Calcium-Calmodulin), Related to Fig. 8, *Ai* and *Aii*

To mimic the action of myosin motors tethered at the PSD, we coated the microscope coverslip with a high-density surface layer of randomly oriented heavy meromyosin (HMM) using protocols from in vitro motility assays. Upon the addition of ATP, CaMKII cross-linked actin filaments remained immobilized, whereas free filaments glided closely juxtaposed to and aligned with the static filaments (47) without becoming fragmented as commonly happens in motility assays performed at high HMM surface densities (48) (Fig. S4 *C*; Video S6). At the same HMM surface density, F-actin networks were not created if calcium-calmodulin was present in the CaMKII/F-actin mixture, and the filaments fragmented upon addition of ATP as reported before (40).

Video S6. HMM Powered F-Actin Gliding along Filaments in an Immobilized CaMKII-Actin Calmodulin), Related to Fig. S4 Cii

## Discussion

Four aspects of CaMKII-actin networks have been explored in our study: 1) the assembly and mechanics of F-actin cross-links formed by CaMKII; 2) the physical role of the CaMKII linker region in network connectivity; 3) the holoenzyme dissociation and network disassembly by calcium-calmodulin, modulated by ATP and tatCN21; and 4) the effect of molecular crowding agents and myosin motors on network architecture and resulting implications for the structural role of CaMKII during early LTP (Fig. 1). Taken together, the results show how CaMKII multivalent linkages form F-actin networks that are sufficiently robust to resist the forces produced by molecular crowding and myosin motors. This suggests that CaMKII-actin networks may maintain the resting morphology of dendritic spines with disruption by calcium-calmodulin, accounting for spine remodeling after synaptic stimulation. The weak monovalent affinity of CaMKII for G-actin allows rapid dissociation by calcium-calmodulin for CaMKII sequestration at the PSD and increase in the G-actin pool, leading to de novo filament formation.

### Assembly and mechanics of cross-linked actin networks

Observed changes of the parameters *Q* and *R* described F-actin network architectures under both steady-state and dynamic experimental conditions. A random network with Poisson-distributed nearest-neighbor junction distances (∼1) was obtained at low CaMKII-actin molar ratios, implying little or no interaction between neighboring nodes. The network was stabilized by preferential localization of CaMKII to junctions. When the ratio of CaMKII to F-actin was increased, F-actin bundles were progressively formed, and network architecture transitioned to an aggregated type (R < 1). The molecular crowding agent, PEG, caused a similar transition by bundling F-actin.

EM images of *β*_Rat_/F-actin documented that junctions are abundant at substoichiometric (0.3) ratios. Published electron micrographs of CaMKII-actin networks show images similar in appearance to ours at comparable molar ratios, whereas bundling dominates (>80%) at equimolar and higher ratios (7). The LM and EM make the case that filaments bundle by being stapled together at multiple points by the holoenzymes in stochastic fashion as concentration increases. The randomness parameter, *R*, introduced in this study provides a ground-level descriptor of model networks to frame theories regarding their assembly and mechanism. It would be of interest to relate this measure to higher-level descriptors of bundle mechanics and viscoelasticity (37, 49) in future work. Here, we found that CaMKII bundles and the bundles formed by crowding agents both have *R* values that decrease from 1 (random) as PEG or CaMKII concentration is increased, which is consistent with the fact that the bundles are aggregations formed by random, isotropic interactions. In contrast, ladder-like bundles periodically spaced by the bivalent actin cross-linker, *α*-actinin (50), form ordered lattice networks (51) that would be predicted to have high *R* values in our assays, as might be tested in the future.

A study (36) of streptavidin/F-actin_biotin_ cross-linked networks mixed with myosin filaments showed that upon ATP addition and myosin filament force production, streptavidin cross-links increased the coalescence of actomyosin foci (0.6 *μ*m/s) from the rate in absence of streptavidin (0.1 *μ*m/s) at low biotin levels (1/1000 biotin/actin molar ratio) but retarded (0.03 *μ*m/s) at fivefold higher levels because of the tension of connecting filament cables opposing motor forces. Here, we found that coalescence of the foci was blocked in the presence of CaMKII. We conclude that under our experimental conditions, CaMKII-actin networks have sufficient mechanical resilience to resist the compaction forces exerted by myosin miniature filaments.

CaMKII-actin networks can complement studies of model systems such as streptavidin-biotin cross-linked actin networks in important ways by exploiting calcium-calmodulin as a tool to dial the affinity and number of cross-links formed. Although the affinity of a single biotin-streptavidin bond (52) is orders of magnitude greater than the *β*rat affinity for G-actin (13), the dodecameric nature of *β*rat compared to tetrameric streptavidin may lead to a smaller difference in binding avidity for F-actin. We note that steric constraints would likely limit the number of binding sites that could attach simultaneously to the same actin filament or to a closely approximated group of actin filaments.

### The role of the linker domain in CaMKII-actin networks

The nematode *d*_*C. elegans*_, with a linker length comparable to the *α*_Rat_ and *γ*_Rat_ linkers, forms networks at equivalent molar concentration, albeit with slightly lower junction density and filaments/junction. This result, together with published results for the rat isoforms (53), shows that much of the *β*_Rat_ linker is not essential for actin binding. The two conserved linker segments identified by the multiple sequence alignment (MSA), the *α*-helical core piece and the unstructured serine/threonine-rich sequence element, may together constitute an actin-binding surface, although the possibility that such a surface is outside the linker cannot be ruled out (15). The additional linker residues may modulate actin binding because the mutation of all autophosphorylation sites to aspartate (a phospho-threonine mimic) in the *β*_Rat_ linker and adjacent residues abolishes F-actin binding; however, mutation to the small amino acid alanine has a small effect (5). Nevertheless, the long-linker *β*_Hum_, whose 220-residue linker is predicted to be largely (>90%) unstructured, forms networks that are indistinguishable from those formed by *β*_Rat_ at similar concentrations; thus, actin binding is not simply proportional to linker length. The persistence length of unstructured peptides is short (∼1 nm) (54), so even short-linker holoenzymes may flex to bind multiple F-actin subunits, with further increase in linker length being inconsequential.

Long linkers may alternatively function to regulate holoenzyme activation instead of facilitating CaMKII-actin network formation. Serine/threonine phosphorylation increases persistence length of unstructured peptides (55) and is a prominent strategy in signaling mediated by phosphorylation-regulated protein-protein interactions (56). Linker extensibility, consistent with an EM rapid-freeze replica (57) and cryotomogram (20) images of the *α*_Rat_ holoenzyme, has rationalized how engineered *d*_*C. elegans*_ six-residue linker insertions or deletions alter the Hill coefficient for substrate phosphorylation (58, 59). Kinase cooperativity has been studied thus far with short (<40 residue) linkers. Investigation of the much longer *β*_Hum_ linker merits future study because it may tune *β*_Hum_ response to calcium stimuli for another purpose and in a different tissue (60).

### Mechanism of calcium-calmodulin-triggered dissociation

The single-molecule evidence for direct GFP-*β*_Rat_ binding to G-actin and F-actin extends conclusions from less direct methods based on intact cell cytoskeletons (14, 17) or correlated fluorescence intensity fluctuations (13). The measured dwell times showed that associations-dissociations occur on the second timescale for most (>90%) of the population. GFP-*β*_Rat_ dissociation from G- to F-actin induced by calcium-calmodulin could not be resolved by flow-cell exchange consistent with the reduction of dwell times. The single-exponential fit for dwell-times obtained for GFP-*β*_Rat_ association with G-actin contrasts with the tailed dwell time distributions obtained for the F-actin networks. The long-lived GFP-*β*_Rat_ holoenzymes trapped at filament junctions or bundles probably account for this difference and may also limit the disassembly (∼1 min) of the in vitro *d*_*C. elegans*_ actin network. The invertebrate *d*_*C. elegans*_-actin network and its calcium-calmodulin-triggered dissociation reported here for the first time, to our knowledge, suggest the utility of CaMKII-actin networks for cytoskeletal signal transduction applies beyond vertebrate cardiovascular and neuronal functions that have traditionally driven CaMKII research.

The rapid phase of GFP-*β*_Rat_ dissociation from F-actin was similar to its dissociation from G-actin, consistent with a common actin-binding interface for F- and G-actin. The unbinding rate, *k*_*off*,_ that occurs after adding calcium-calmodulin is similar to that measured by TIRFM SPT in cultures of human umbilical vein endothelial cells (*k*_*off*_ = 6.5 s^−1^) for the GFP-*β*_rat_ T287D mutant that does not bind cytoskeletal actin (14). The refractory fraction resistant to calcium-calmodulin alone was dissociated when calcium-calmodulin was supplemented by tCN21. This result implies that the peptide tCN21 binds to individual subunits opened by calcium-calmodulin to shift the inactivatable holoenzyme forms toward extended, activatable states. In contrast, ATP did not reduce the refractory-bound fraction probably because the ATP binding site is not fully formed in the autoinhibited conformation. A similar rationale was used to explain why the autocamtide-2 inhibitor peptide, an autophosphorylation site mimic, did not affect dissociation (5).

The calcium-calmodulin responsive and refractory subpopulations documented in our single-molecule experiments matched the estimates for the extended versus compact autoinhibited holoenzyme conformations seen in cryoelectron tomograms (20). The match is consistent with binding of calcium-calmodulin to the extended conformation alone. The fact that calcium-calmodulin disassembles networks within 1 min indicates that the refractory populations either do not block network disassembly or transition over a minute to the extended, calcium-calmodulin responsive conformation. Thus, calmodulin access is not a concern for early LTP.

### Implications for early LTP

Cytosolic calcium increases rapidly to millimolar levels after synaptic stimulation because of the inward NMDA calcium current to initiate the transition from state (*i*) to state (*ii*) (Fig. 1 *C*). Rapid (a few seconds) dissociation of CaMKII from both F-actin and G-actin, as reported here, has a twofold effect: polymerization of the released G-actin that can proceed immediately and slower disassembly of the CaMKII-actin network. The latter would coincide with the time course of myosin-II polymerization into miniature filaments that would be slow because phosphorylation must precede myosin filament assembly. Thus, our results are compatible with the scenario in which the rapid initial phase of spine expansion is due primarily to actin polymerization, whereas subsequent spine compaction occurs as polymerization slows and myosin motor activity increases. Although the calcium transient is short lived, the newly formed myosin miniature filaments as well as the PSD-sequestered, NMDA-bound CaMKII will continue to be active (61, 62).

The molar ratio of CaMKII/G-actin in the basal state of hippocampal neuron dendritic spines is around 0.3 (Fig. 1, state *i*). This estimate is based on the CaMKII concentration (138 *μ*M) (8) and assumes actin is comparable to lamellipodia (500 *μ*M as F-actin out of 650 *μ*M total) (63). We find that <25% of F-actin is bundled at this molar ratio, but bundling occurs at higher molar ratios. The basal dendritic spine free-calcium level is subnanomolar, so calmodulin is in its apo form, and CaMKII-actin networks are stable and not susceptible to forces due to macromolecular crowding or episodic myosin motor activation triggered by miniature synaptic currents (64). It is not presently known whether myosin motors act as individual molecules tethered to the PSD or as a collective by assembling into miniature filaments. Our assays recreated both scenarios with myosin motors at higher concentrations than likely to obtain at the synapse. Myosin-II activity at the leading edge is thought to fragment actin networks for turning in neuronal growth cones (27). Neurons express the *β*_E_ splice variant that lacks actin-binding functionality during early development (12); thus, immature spine filopodia may also utilize myosin-induced fragmentation unconstrained by CaMKII cross-links to orient to developmental cues. Fragmentation will, however, be deleterious for mature spines once synapses form, so CaMKII-actin networks may be important blockers of episodic myosin activity. The CaMKII-actin networks in our assays were resistant to both myosin-powered compaction and fragmentation.

The mechanical strength of the CaMKII-actin network is an advantage under basal conditions to counteract physical force but a disadvantage when the same forces are needed to remodel the spine. Thus, if CaMKII-actin networks maintain spine size under basal conditions, it follows that synaptic stimulation must couple CaMKII dissociation to network disassembly. The single-molecule TIRF experiments establish that the dominant actin-bound GFP-*β*_rat_ fraction (∼80%) dissociates within seconds, which is consistent with this premise. The activated, dissociated holoenzymes will retain the extended conformation with rebinding of R_1_ to its parent subunit blocked by subunit capture and calmodulin trapped by T287 phosphorylation (65). The stable end state (Fig. 1 *C*, state *iii*) for spine volume is reached after 3 min. The myosin miniature-filament-powered compaction reported in this study is rapid enough to account for the compaction from the peak to the end-state volume of the stimulated spine. On the other hand, compaction due to clustering mediated by crowding agents may be ruled out because it is too slow. As the new basal state is established, the actin cytoskeleton will again be stabilized by CaMKII cross-linking.

Our data show that tCN21 facilitates dissociation from F-actin. It may do so by shifting the holoenzyme conformational equilibrium to the extended form upon binding subunits opened by calcium-calmodulin. Regardless of mechanism, this result implies that CaMKII associated with NMDA receptors is mechanically uncoupled from cytoskeletal actin. The uncoupling may prolong PSD-sequestered CaMKII activation to allow PSD remodeling to be completed. However, CaMKII interactions with other cytoskeletal proteins, notably *α*-actinin (66), might be as important, if not more so, to synchronize the changes in cytoskeletal and PSD architecture for increased synaptic strength.

A model (28) based on transcriptome analysis (67) and live-cell imaging of fluorophore-tagged proteins (kinases: RhoA, Cdc42; actin-binding proteins: cofilin/Arp2/3) (23, 68) simulated CaMKII-mediated phosphorylation cascades for actin polymerization and subsequent compaction by myosin motors. The model accounted for the observed changes in spine volume (Fig. 1 *C*) that were robust to rate and concentration variations as assessed by sensitivity analysis (69). The current study characterizes the important role of CaMKII in regulating cytoskeletal architecture. This idea was implicit in but not explicitly addressed by the model. Rapid disassembly of the actin cytoskeleton, in particular the CaMKII-actin networks described here, must be coupled to CaMKII dissociation by calcium-calmodulin to allow myosin-powered F-actin network compaction to proceed. Spine remodeling could be fine-tuned by fragmentation of the disassembled actin filaments by PSD-tethered myosin motors for polymerization at the spine tip. Thus, CaMKII could serve as a synaptic tag but in a profoundly different sense than proposed by Lisman (70) and others (71) in that it orchestrates stimulus-induced volume changes of the dendritic spine and more importantly maintains the volume difference between stimulated and nonstimulated spines over times long enough to initiate downstream processes for LTP.

In conclusion, this study provides a novel, to our knowledge, view of CaMKII-actin network architectural dynamics to better define the structural role of CaMKII that has been largely considered thus far in terms of its association with the PSD (70). The architecture is remarkable in that it seems to have evolved to both fix spine size over hours under basal conditions as well as disassemble in response to a calcium-calmodulin pulse, within a minute, to complement its kinase activity during early LTP. Measurements of the calcium-calmodulin-triggered network disassembly and its modulation by ATP and membrane receptor targets have mechanistic implications that may be tested and refined by numerical simulations and in vivo experiments.

## Author Contributions

S.K. designed/performed experiments, analyzed data, and wrote the manuscript. K.H.D. designed/participated in EM experiments and edited the manuscript. J.E.M. designed experiments, analyzed data, and wrote the manuscript.

## Supporting Citations

References (72, 73, 74, 75, 76, 77, 78, 79, 80, 81, 82, 83) appear in the Supporting Material

## Supporting Material

Document S1. Supporting Materials and Methods and Figs. S1–S4

Document S2. Article plus Supporting Material
