# Supplementary material for: Architectural Dynamics of CaMKII-Actin Networks
Source: Biophys J. 2018 Nov 10;116(1):104–19. doi: 10.1016/j.bpj.2018.11.006 (PMC6341221; doi:10.1016/j.bpj.2018.11.006)
Supplement: Document S1. Supporting Materials and Methods and Figs. S1–S4 [file mmc1.pdf]

Biophysical Journal, Volume *116*

Supplemental Information

Architectural Dynamics of CaMKII-Actin Networks

Shahid Khan, Kenneth H. Downing, and Justin E. Molloy

SUPPORTING MATERIAL

A. Protein Chemistry

Bioinformatics: CaMKII sequences were downloaded from Uniprot (76) and clustered with CD-Hit (77). MUSCLE (78) was used for multiple sequence alignment (MSA). The MSA was manually curated in Jalview (79). Phylogenetic trees were constructed with FastTree 2.19 (80) and displayed with FigTree 1.4 (*http://tree.bio.ed.ac.uk/software/figtree/*). Linker length variation was a dominant factor in the greater spread of the linker versus the KD phylogenetic tree. The increased diversity in the vertebrate populations may be a consequence of gene duplication (**Figure S1A**).

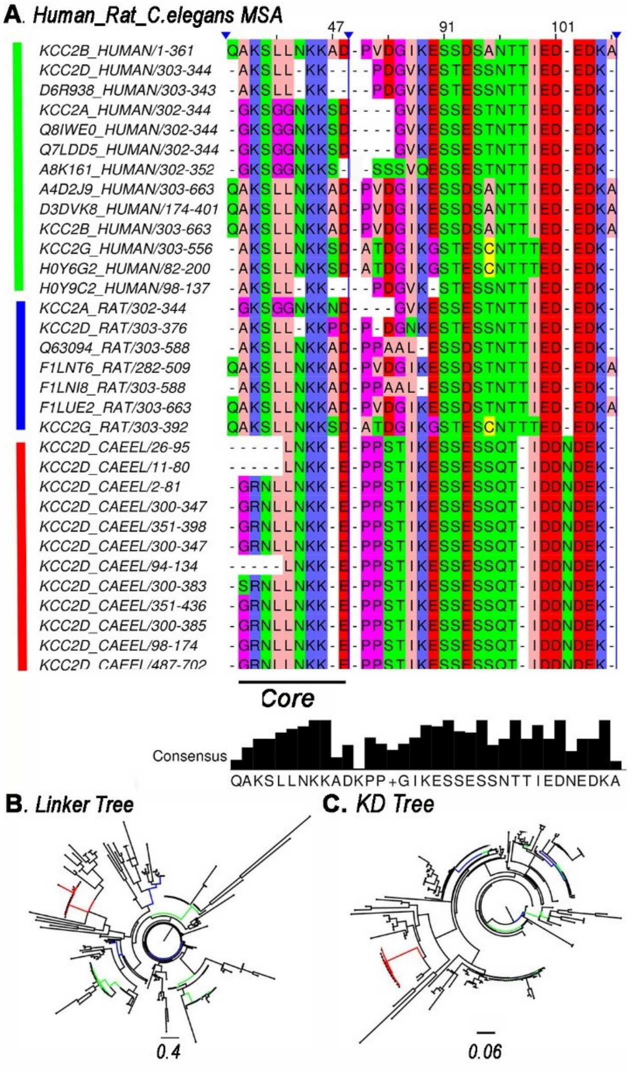

***Figure S1: Linker domain conservation & phylogenetics. A.** MSA of the two conserved segments in linker sequences from > 20 human (green bar), rat (blue bar) and C. elegans (red bar) CaMKII isoforms and splice variants. Residues are coloured according to type (JalView – Zappo). Core (black line) marks the core linker sequence reported in (39). Domain phylogenetic trees constructed with sequences from > 500 CaMKII isoforms / splice variants from Uniprot database reveal the variability of the linker (**B**) relative to the kinase domain (**C**). The C. elegans, but not the rat or human, sequences are monophyletic.*

Disorder profiles were generated with DisoPred3 (49). DisoPred3 recognizes intrinsically disordered regions based on evolutionarily conserved residues that are missing or have high temperature factors in high-resolution X-ray structures deposited in the Protein Data Bank (81) database. PsiPred (82) was used for secondary structure prediction. to identify unstructured (disordered) regions that may allow kinase domains to extend out from the hub. The 90-residue linker together with 40 N-terminal AD residues is the most unstructured part of the β_{Rat} protein. The shorter linker rat isoforms had similar disorder profiles over over-lapping sequence segments. The entire β_{Hum} linker segment subunit (>100 residues) additional to the β_{Rat} linker, was predicted to be unstructured. The known crystal structure (KD+AD) of the β_{Hum} subunit (with unknown linker structure) was used to check our secondary structure predictions and the structured regions were superimposed on the disorder profiles. An α-helix was predicted with high confidence for the core peptide, but the other conserved peptide was unstructured (**Figure S1B**).

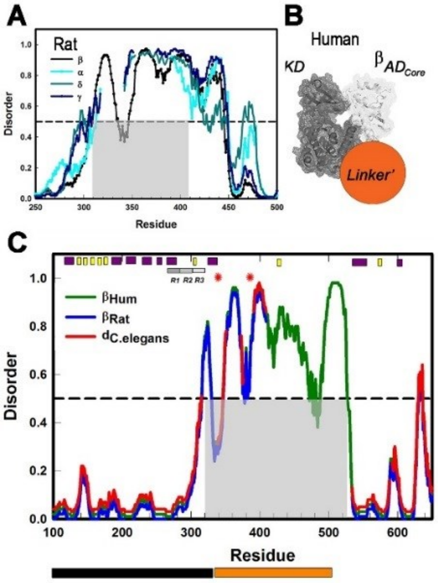

***Figure S2: Linker domain disorder profiles: A.** Disorder profiles of the rat isoforms from their MSA**.** Shaded box marks the 90-residue "actin binding domain" linker. About 40 N-terminal AD residues are also predicted to be unstructured. **B.** Human CaMKII subunit (3SOA.pdb) with superimposed linker size estimated from Stokes radius. **C.** Disorder profile from the MSA of the nematode ($d_{C.elegans}$, red), rat*

(β_{Rat}, blue) and human (β_{Hum}, green) CaMKII subunit sequences. Horizontal bars mark predicted secondary structure (magenta (α-helix); yellow (β-sheet)). There was good agreement between the predicted secondary structure and the crystal structure; although the KD R2 / R3 helix seen in the crystal was not predicted consistent with EPR measurements (83). Asterisks mark conserved linker segments. Black and orange bars mark the β_{Hum} kinase and extended linker (linker') domains respectively. Shaded box includes the additional 40 AD residues. N -> C residue numbers. Values > 0.5 (dashed lines) indicate significant intrinsic disorder.

Protein Purification: The Rosetta BL21 / DE3 bacterial system (Novagen) designed to enhance expression of eukaryotic proteins was used for plasmid encoded expression of CaMKII and calmodulin. Protein phosphatase was co-expressed from a compatible plasmid (69). After sonication and removal of cell debris in the presence of a protease inhibitor cocktail (Sigma-Fast S8830) CaMKII holoenzymes were purified by elution from a nickel column (5 ml Ni-NTA Hi-trap), protease cleavage and desalting, flow through again through the nickel column followed by anion exchange (1 ml mono-Q Hi-trap) and subsequent gel filtration (sephacryl S-500) following published protocols (53). Calmodulin was purified, after sonication and removal of cell debris as above, with a CL4B phenyl-sepharose column (71). All columns were purchased from GE Healthcare. Western blots of β_{Rat} were based on ECL detection (BioRad #1705061) of its endogenously biotinylated Avi tag; double-stained with horseradish-peroxidase conjugated streptavidin (Thermo-fisher Scientific) and Ponceau-S protein stain. Proteins were transferred from gel (4-15% polyacrylamide) to membrane (PVDF) with the Criterion blotter system (Bio-Rad #170-4070). Proteolysis at multiple sites in the β_{Rat} linker domain was observed when preparations were left on ice for a prolonged period (>1 day) (**Figure S3A**).

Cos7 cell cultures were transfected with plasmids encoding native and mutant GFP-β_{Rat} proteins as reported previously (15). Lipofectamine-2000 was used to co-transfect plasmids encoding phosphatase together with plasmids encoding CaMKII constructs. There was no measurable co-localization of green fluorescence in extracts from non-transfected Cos7 cultures in control experiments. Extracts were typically diluted 50 to 100-fold in AB^-, but 10-fold lower dilution did not affect results. Clarified Cos7 cell extracts of GFP- β_{rat} was prepared, flash-frozen and stored at -80°C following (16). Extracts were used within 24 hours, once thawed, to minimize any GFP- β_{rat} aggregation.

Actin & Myosin Biochemistry. Actin, myosin, and the proteolytic sub-fragment of myosin called heavy mero-myosin (HMM) were prepared from rabbit back and leg fast skeletal muscles by established protocols ((44) and references therein). Myosin was stored at -20° C as a 50% glycerol stock solution. One ml aliquots of G-actin (4 mg/ml) were stored at -80° C. Myosin filaments were prepared after one cycle of

polymerization / sedimentation / depolymerization by rapid dilution from 0.5 M to 0.1 M KCl following (72). Cy3-actin was prepared by incubation of 3-5 mg of G-actin with Cy3 maleimide (GE Healthcare). The labelled G-actin was separated from dye with a Sephadex-G25 column and the dye / protein ratio in the protein fraction determined following the manufacturer's instructions.

Negative-stain electron microscopy: Phalloidin stabilized F-actin (3 μM) with CaMKII in AB^- buffer solution (100 μl) was incubated on inverted 200-mesh carbon coated grids (Ted Pella (Redding, CA)) in a humidity chamber for 30 minutes at ambient temperature, washed twice in 10 mM Tris pH 7.0, then stained with 2% uranyl acetate. Images were acquired at 80 kV with a Gatan MegaScan 794/20 camera mounted on a JEOL 1200CX electron microscope.

B. MICROSCOPY & IMAGE ANALYSIS

TIRFM: Five different laser lines (405nm, 488nm, 532nm, 561nm, 635nm) were combined using a series of dichroic mirrors and co-aligned along a single path that was incident at the Bragg angle of an acousto-optical tuneable filter (AOTF, MDSnC, AA Sa Opto-Electronic Division, Orsay, France). Different laser lines were deflected to their 1st order diffraction position using a radio frequency driver amplifier to produce travelling acoustic waves in the AOTF medium. The selected laser-lines then passed through an 8x beam-expander, using a pinhole to reject the unwanted zero order laser beam paths. The selected laser beams were then directed into the microscope epi-fluorescence light path using a system of mirrors and lenses mounted on a combination of translation mounts and kinematic mounts (Thorlabs Inc., Newton NJ, USA). The light was brought to a focus at the extreme edge of the back aperture of the objective lens (Nikon, 100x TIRF 1.45NA) so that it exited the objective as a parallel beam at an angle to the object plane exceeding the critical angle (63°) requirement for total internal reflection at the coverslip / water interface (i.e. specimen). The microscope stage and objective lens employed piezo-positioners to control specimen position and image focus. Images were acquired with an EMCCD camera (96 nm/pixel magnification).

Biotinylated F-actin filaments were attached to streptavidin-coated coverslips as described for the epi-fluorescence experiments with the difference that unbound filaments were washed out after 3 rather than 30 minutes. AB^-/GOC with GFP-β_{Rat} was then flowed in.

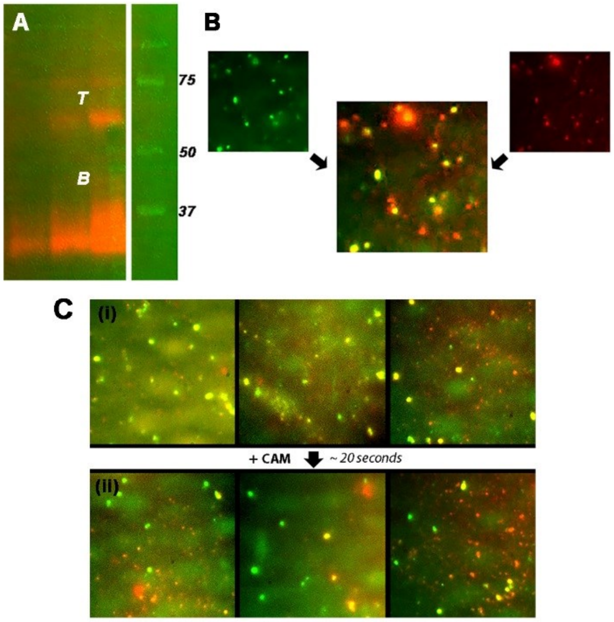

Figure S3: A. CaMKII Western blot. *Streptavidin (red) bound to the N-terminal Avi tagged β_{Rat} (62.8 kD) and Ponceau S protein stain (green). MW markers are labelled. The Avi tagged bands are 63 kD (T) and 33.1-36.7 kD (B). The lower bands (B) are compatible with the kinase domain (36.5 kD).* ***B. Single molecule G-actin association / dissociation with immobilized holoenzymes. (****Colocalization of Cy3 G-actin with antibody immobilized βrat. 50-frame average. Scale Bar (white) = 2 μm.* ***C.*** *Image fields from an experiment comparing Cy3 G-actin co-localization in the absence (0.33 $\pm$ 0.08 Pearson's correlation)) and presence ((0.015 $\pm$ 0.04 Pearson's correlation)) of calmodulin in Ca^{2+}.AB^- buffer. The latter images (50 frame average) were recorded immediately after introduction of calmodulin.*

Epi-fluorescence Microscopy: For crowding assays, rhodamine-phalloidin F-actin was mixed with PEG AB-/GOC solutions. The mixtures were perfused into BSA coated flow chambers. Chamber depth was controlled by 2.3 μm silica beads (Bang Laboratories, Fishers, IN). For gliding assays, HMM was bound to the nitrocellulose-coated coverslip by flowing 25 μl of 200 μg/ml of HMM in AB^- into the flow cell and incubating for 1 minute. After 2x wash, CaMKII / Rh-Ph F-actin mixtures were perfused in and incubated for 30 minutes. Unattached filaments were washed out before addition of AB^+/GOC. For compaction assays, the glass coverslip was coated with streptavidin, blocked with BSA. Synthetic F-actin filaments polymerized with biotinylated G-actin (Cytoskeleton Inc., Denver CO, USA) in 1:50 ratio with G-actin and labelled with rhodamine-phalloidin (Rh-Ph F-actin$_{biotin}$) were flowed in after extensive (4x) washing with AB^- buffer. Then, CaMKII / Rh-Ph F-actin mixtures perfused in and incubated as above.

LM SPT & Image Analysis. Single β_{Rat} molecules, imaged with TIRF, 1 μm polystyrene beads, imaged by bright-field illumination or centroids of actin cables were tracked. Lateral diffusion coefficients (D_{lat}) were calculated from the mean square deviation (MSD) with either automated single particle

tracking (ASPT) or manual tracking GMimPro functions. D_{lat}'s for single bead tracks were used for estimation of the viscosity of PEG solutions (**Figure S4A (i)**).

ImageJ plugins were used for kymograph and colocalization analyses. Kymograph analysis combined manual measurements of spots with lifetimes > 0.3 s (6 frames) with automated spot detection, with Particle Analysis function for spots with short lifetimes (< 0.3 s) with intensity threshold and subsequent lifetime determination by division of the spot area by kymograph unit area (1 frame * 1 pixel). For colocalization analysis binary masks of the GFP-β_{Rat} spots generated based on the intensity threshold were applied to both channels (GFP-β_{Rat} (green), Cy3-actin (red)) to record the intensity overlap and the Pearson's correlation score.

The SOAX persistence lengths, l_p, fitted the assumed worm-like chain model well (**Figure S4A (ii)**), but were systematically under-estimated by a factor of two compared to the literature value (16.2 μm) for single F-actin filaments (74), similar to underestimates for l_p determined for single microtubules computed by SOAX (46).

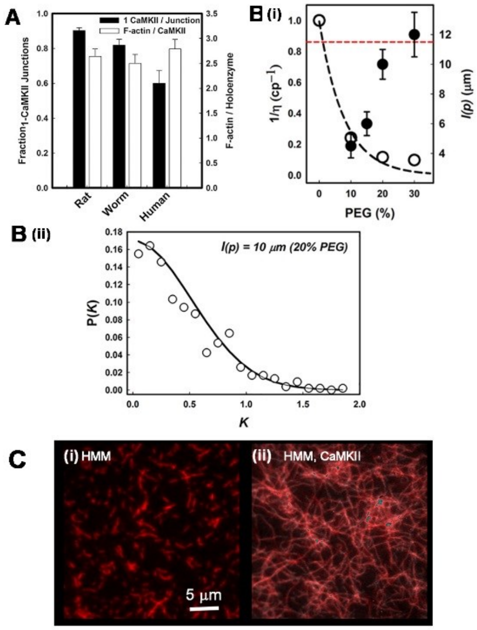

Figure S4: A. Comparison of CaMKII-actin networks. *The junction density for the* $d_{C.elegans}$ *networks was lower (31.3±2.1 /* μm^2*) relative to* β_{Rat} and β_{Hum} *networks (50±4.2 /* μm^2*).* ***B. PEG F-actin suspensions. (i)*** *The relative viscosity (open symbols) is plotted from the* D_{lat} *measured for 1* μm *polystyrene spheres (n = 20). For* η *= 1 cp (0% PEG),* η *= 4.1, 8.6 and 10.2 cp for 10%, 20% and 30% PEG solutions respectively. The reported viscosity for mammalian cell cytoplasm is 3.2 cp (73). It may be as high as 10 cp in dendritic spines that are more akin to the cell cortex (50). Persistence lengths of actin cables*

(closed symbols) are also shown. ***Horizontal dashed red line marks the persistence length obtained with*** *$d_{C.\ elegans}$ in Figure 6Ci.* ***(ii)*** *Fit of the 2D worm-like model to the 20% PEG (K) distribution.* ***C. F-actin fragmentation by surface-immobilized HMM. (i)*** *F-actin filaments $(\langle l_x \rangle = 1.5\ \mu m)$ gliding on an HMM coated surface. The gliding filaments align when crowded.* ***(ii).*** *F-actin filaments gliding (silver outlines) on an HMM coated surface along immobilized filaments (red). $(\langle l_x \rangle = 2.5\ \mu m)$ cross-linked by $d_{C.elegans}$. Gliding was initiated after 30-minute incubation on HMM coated glass by flow-in of AB^+/GOC. Scale as for A.*

In the absence of ATP, mixtures of $d_{C.elegans}$ CaMKII and F-actin (at 1:3 molar ratios) formed a contiguous, interconnected filament network that attached to HMM-coated surfaces. The network did not disassemble when ATP (AB^+) was perfused in (**Figure 5 A, B**). A sub-population of filaments that was not part of the network aligned (41) and glided along rails of static filaments in the immobilized network (**Supporting Material Movie S4).** When calcium-calmodulin was introduced networks disassembled over a period of ten minutes and dissipated as the HMM translocated the separated filaments.

Supporting Videos (for **Figures)**

Movie M1: *Figure 2A. SOAX representation of a F-actin network.*

Movie R1: *Figure 4Aii,5Aii. TIRFM of* GFP-β_{rat} *association / dissociation with F-actin ((+/-) calcium calmodulin).*

Movie R2: *Figure 4C-5Di. TIRFM of* GFP-β_{rat} *association / dissociation with G-actin ((+/-) calcium calmodulin).*

Movie R3*: Figure 6C-D. C. elegans CaMKII-actin, network ((+/-) calcium calmodulin).*

Movie R4: *Figure 8Ai, ii. Myosin mini-filament powered compaction ((+/-) CaMKII, (+/-) calcium calmodulin)*

Movie S1: *Figure S4Cii. HMM powered F-actin gliding along filaments in an immobilized CaMKII-actin network.*
